# What Makes a Potent Nitrosamine? Statistical Validation of Expert-Derived Structure–Activity Relationships

**DOI:** 10.1021/acs.chemrestox.2c00199

**Published:** 2022-10-27

**Authors:** Robert Thomas, Rachael E. Tennant, Antonio Anax F. Oliveira, David J. Ponting

**Affiliations:** Lhasa Limited, Granary Wharf House, 2 Canal Wharf, LeedsLS11 5PS, United Kingdom

## Abstract

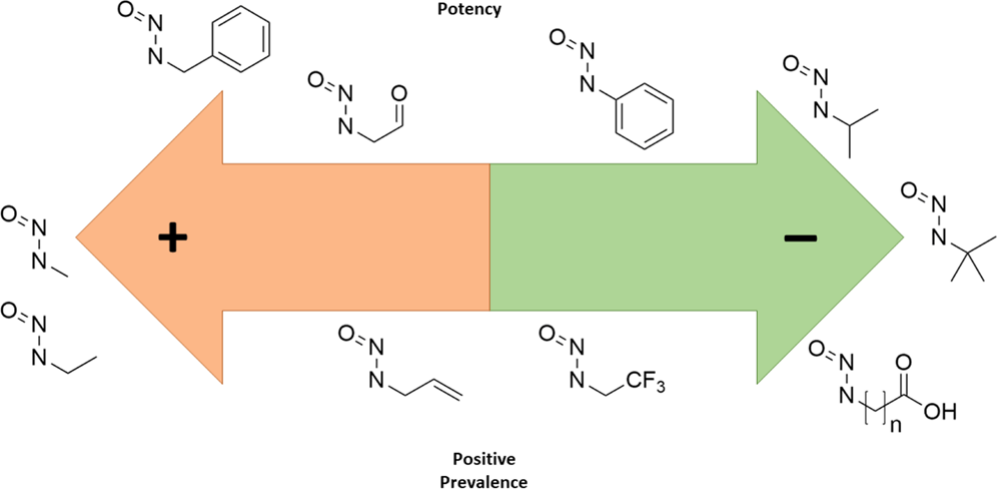

The discovery of carcinogenic nitrosamine impurities above the safe limits in pharmaceuticals has led to an urgent need to develop methods for extending structure–activity relationship (SAR) analyses from relatively limited datasets, while the level of confidence required in that SAR indicates that there is significant value in investigating the effect of individual substructural features in a statistically robust manner. This is a challenging exercise to perform on a small dataset, since in practice, compounds contain a mixture of different features, which may confound both expert SAR and statistical quantitative structure–activity relationship (QSAR) methods. Isolating the effects of a single structural feature is made difficult due to the confounding effects of other functionality as well as issues relating to determining statistical significance in cases of concurrent statistical tests of a large number of potential variables with a small dataset; a naïve QSAR model does not predict any features to be significant after correction for multiple testing. We propose a variation on Bayesian multiple linear regression to estimate the effects of each feature simultaneously yet independently, taking into account the combinations of features present in the dataset and reducing the impact of multiple testing, showing that some features have a statistically significant impact. This method can be used to provide statistically robust validation of expert SAR approaches to the differences in potency between different structural groupings of nitrosamines. Structural features that lead to the highest and lowest carcinogenic potency can be isolated using this method, and novel nitrosamine compounds can be assigned into potency categories with high accuracy.

## Introduction

Recent discovery of nitrosamine impurities in marketed drugs has led to a rapid evolution of regulatory activity^1−4^ and, in response, analysis of the synthetic and formulation pathways for existing drug products (DPs) as well as novel active pharmaceutical ingredients (APIs) and DPs. Due to the extreme carcinogenic potency^5,6^ of some nitrosamines such as nitrosodiethylamine (NDEA), these compounds are considered to be in the cohort of concern,^7−9^ and a class-specific acceptable intake (AI) of 18 ng/day has been set by the European Medicines Agency (EMA) and other regulators—based on the 5th percentile of known nitrosamine TD_50_ values (the dose that induces tumors in 50% of animals over control, which can be extrapolated to a standardized AI for humans). Read-across to the harmonic mean TD_50_s of NDEA (26.5 mg/kg/day) and NDMA (96 mg/kg/day), corresponding to AI limits of 26.5 and 96 ng/day, respectively, has been proposed for a number of common nitrosamines by the EMA,^1^ U.S. Food and Drug Administration (FDA),^4^ and others. However, the carcinogenic potencies of nitrosamines span a range of at least 4 orders of magnitude,^10^ and these class-based AI limits can be increased^1,4^ not only for those compounds that have reliable carcinogenicity data but also those for which a structurally close analogue with reliable carcinogenicity data can be determined.

This, however, raises the question of “what is structurally similar?”. One approach for structural similarity that is often used is the Tanimoto coefficient of similarity, calculated for the whole molecule; however, this by itself would be a poor method to use for nitrosamines since the carcinogenic potential is critically dependent on the metabolic potential,^11−13^ which is itself dependent on the local environment around the nitrosamine substructure.^12−14^ Approaches have been made subjectively to address nitrosamine structure–activity relationships (SAR);^12−14^ however, the step from “this feature may affect potency” to “this feature has a statistically significant effect on potency” has hitherto not been made for nitrosamines. This work presents a method by which that can be performed. In addition, a comparable method is used for the classification of features as to whether they have an impact on if the nitrosamine is carcinogenic or not (positive prevalence). These two models are referred to as the “regression” and “classification” models henceforth.

While the cohort of concern was defined^7−9^ based on the *N*-nitroso substructure (N–N=O) and thus can be considered to include all *N*-nitroso compounds (NOCs), the main focus of both SAR work and regulatory attention has been on dialkyl nitrosamines—as opposed to nitrosoureas, nitrosoamides, and others (as defined in Figure 2 in Cross and Ponting^14^). These have been observed to have comparable potency to dialkyl nitrosamines but have different requirements for metabolic activation. Results are presented here for analysis performed both on the entire set of *N*-nitroso compounds and considering the subset of dialkyl nitrosamines alone (henceforth referred to as “NOC” and “nitrosamine” datasets).

We have previously shown^15^ that the carcinogenic potencies of *N*-nitroso compounds and nitrosamines as classes of compounds follow a log-normal distribution, and that the same can be said of the various subclasses proposed in that work. Subsequent research by a collaborative cross-industry working group^14^ has refined the potential structural features to provide a list of over 80 features, encoded as SMARTS (SMILES (Simplified Molecular-Input Line-Entry System) Arbitrary Target Specification) patterns. In this work, we present the synthesis of these two previous aspects—statistical methods are used to show that a number of expert-derived features have statistically significant effects on the carcinogenic potency and prevalence of nitrosamines. Furthermore, the statistical analysis of the impact of the features was compared with an independent subjective assessment, performed by an expert in SAR analysis previously uninvolved with this work but familiar with nitrosamine safety assessment.

**Figure 1:**
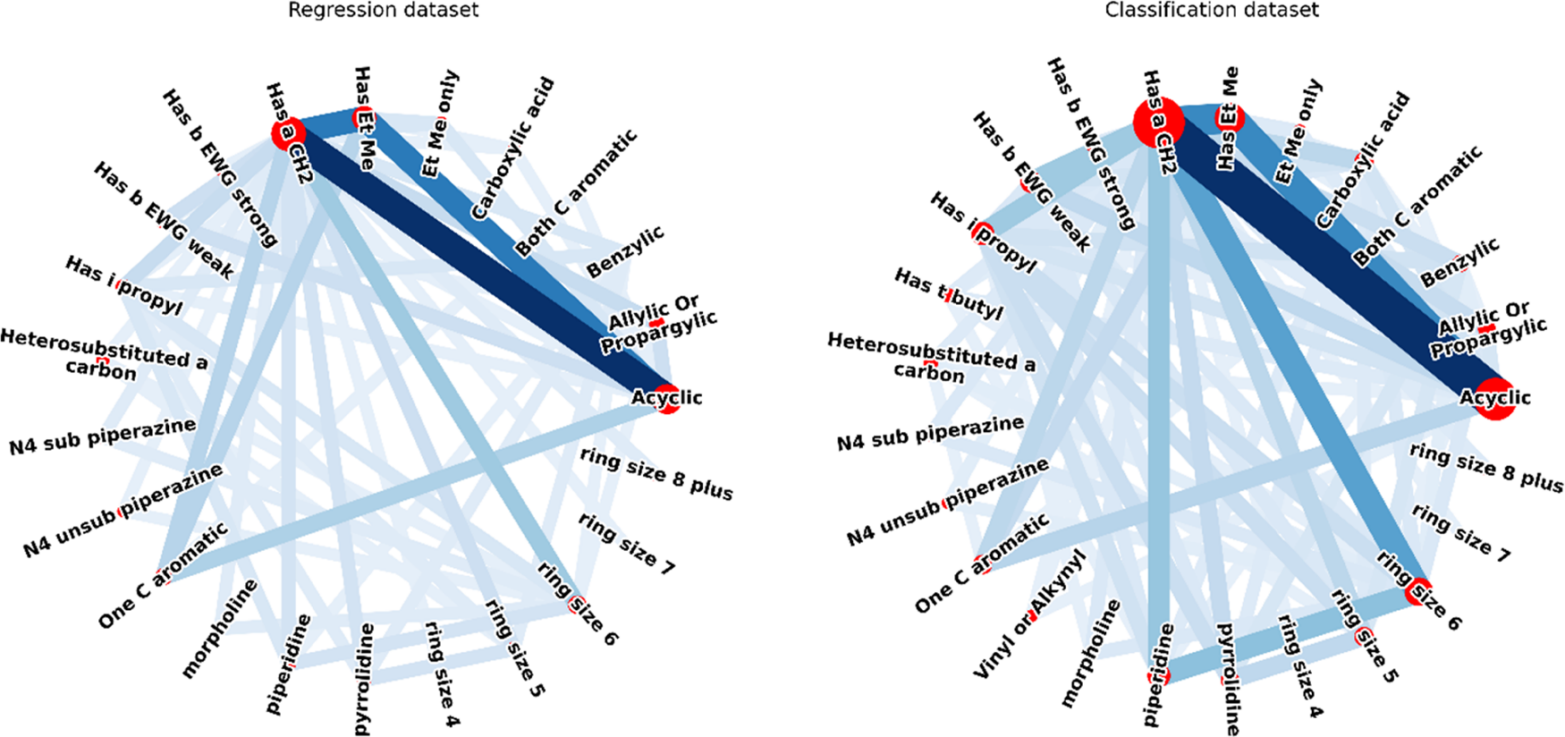
Overlap of features within the available dataset. The width and color intensity of a line is proportional to the number of compounds in the dataset that share a pair of features. The shared features form a complex network of dependencies that must be accounted for.

A key complexity in moving from expert assessment to statistically significant results, which this work seeks to address, is that any given nitrosamine is likely to be a member of multiple substructual categories. For example, *N*-nitrosonornicotine (NNN, see Figure 8b) is a pyrrolidine ring, with an isopropyl-like α-carbon, which is also benzylic—and the different features may have a variety of effects that may variously increase or decrease potency. These may also mask the effect of each other, especially in the relatively small dataset that is available for nitrosamines. The deconvolution of these requires a statistical technique (discussed subsequently) that is able to take dependencies in the data into account and precludes analysis of individual features in isolation. Figure 1 shows, using the set of features described subsequently, the overlaps between categories for the dataset of nitrosamines with available carcinogenicity data. These methods could also be applied to other complex structural classes (e.g., aromatic amines), once an expert-derived list of potentially impactful features is created. Returning to the question of defining the relevance of an analogue for potential read-across to a novel nitrosamine compound, the presence or absence of particular features should be evaluated, especially those shown to have a statistically significant impact on the potency.

## Methods

### Data Curation

**Figure 2:**
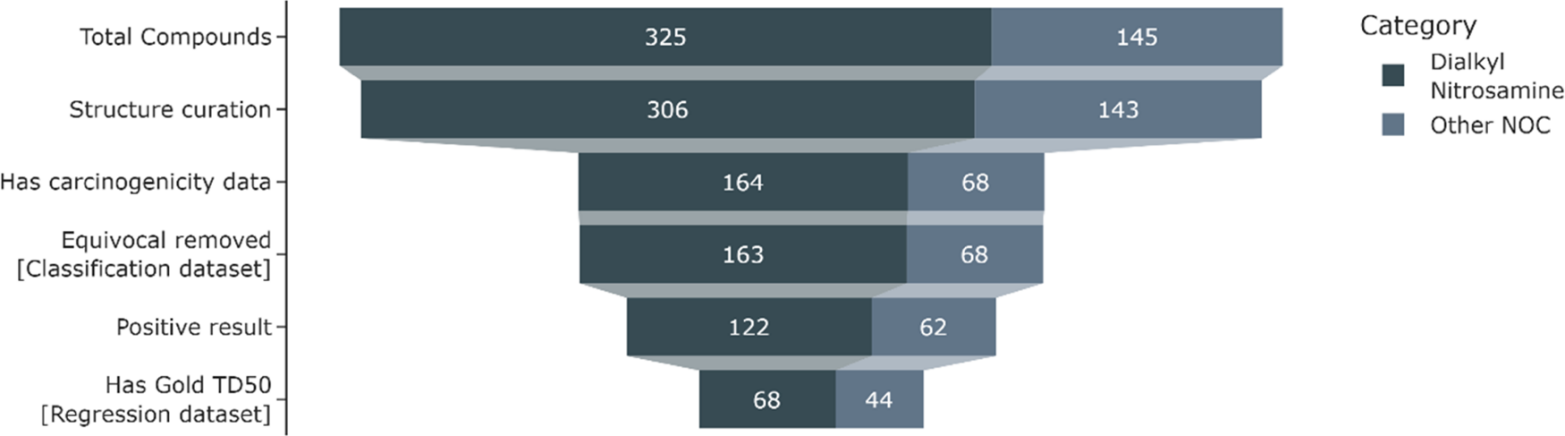
Data curation funnel.

The Lhasa Limited Vitic^16^ database (version 2022.1) contains data for 470 NOC with at least some Ames or rodent carcinogenicity data. These were then filtered to exclude those compounds containing deuterium atoms (to avoid duplicating entries where a series of compounds with identical scaffolds but differing sites of deuteration were used for mechanistic studies), those with multiple nitroso groups and those with an overall call of “equivocal,” resulting in a NOC dataset containing 231 compounds with carcinogenicity data, of which 112 had Gold TD_50_ data in the Lhasa Carcinogenicity Database (LCDB)^17^ (i.e., that calculated according to the method of Peto et al.^6^—the use of Lhasa TD_50_ was considered, but fewer data points are available; where both are available the correlation is exceptionally high^5^). Filtering to only those compounds which match the nitrosamine pattern (the *N*-nitroso group must be bonded to two carbon atoms, neither of which can be doubly or triply bonded to heteroatoms), these numbers become 163 and 68, respectively. The smaller size of the regression, as opposed to classification, dataset arises from two sources: First, the regression model is only trained on positive compounds, so by definition does not contain compounds with negative or equivocal results. Second, there is a proportion of these positive results which were not included in the Carcinogenicity Potency Database (CPDB)^18^ and thus have no TD_50_ value calculated; either the study was simply not incorporated or, while the study is sufficient to identify a positive result, insufficient details were provided to calculate a TD_50_ or the study itself was deficient such that numerical results cannot be extrapolated. Figure 2 shows this breakdown of the data.

### Features

Over 80 features and combinations thereof were developed by Cross and Ponting as SMARTS patterns;^14^ however, a majority of these are combinations and the feature set can be reduced to a set of 41 features that are as close to independent as possible without losing information (i.e., there are some cases where overlap between two features has been permitted, such as “has Et/Me group” and “only Et/Me groups”, and “one aromatic carbon” and “both carbons aromatic”) where these combinations have implications for the mechanism of action. These have been reimplemented into the Lhasa Limited cheminformatics codebase (as Derek^19−21^ patterns) and this set falls into a few main categories:

- Type of *N*-nitroso compound: as discussed briefly above, there are *N*-nitroso compounds of comparable potency to dialkyl nitrosamines but with potential alternative mechanisms of action.^14^
- Degree of steric bulk at the α-carbon.^14^ This covers both steric restriction (such as the presence of isopropyl groups) and the prevention of α-hydroxylation (such as *tert*-butyl groups or aromatic systems).
- Electron-withdrawing potential at the β-carbon.^14^
- Unsaturation close to the α carbon—allylic, propargylic, benzylic, and similar systems.
- Size of ring system—patterns for each of 4, 5, 6, 7 and a group for rings of larger than eight atoms were added; these sizes of rings (as also 3, but no data for nitrosoaziridines exists) may have a significant impact on the reactivity of the nitrosamine group, rather than simply considering cyclic/acyclic as a binary choice.
- Nature of ring system (for common five- and six-membered ring systems such as piperidine). For some classes of common ring, sufficient data exists that these can be considered in their own right rather than a combined consideration of all five- and six-membered rings. This may well assist in refining the SAR, since there is a 15-fold difference in summary TD_50_^5,6^ between the otherwise-similar compounds nitrosomorpholine and nitrosopiperazine, and 18-fold between nitrosopiperidine and nitroso-1,2,3,6-tetrahydropyridine (Table 1, data from the LCDB^17^). Chemical reasons for these differences will be discussed later. Potency values for all nitrosamines have been observed to cover 4 orders of magnitude;^10^ the fact that these four otherwise-similar compounds span more than 2 orders of magnitude themselves is significant!

**Table 1:**
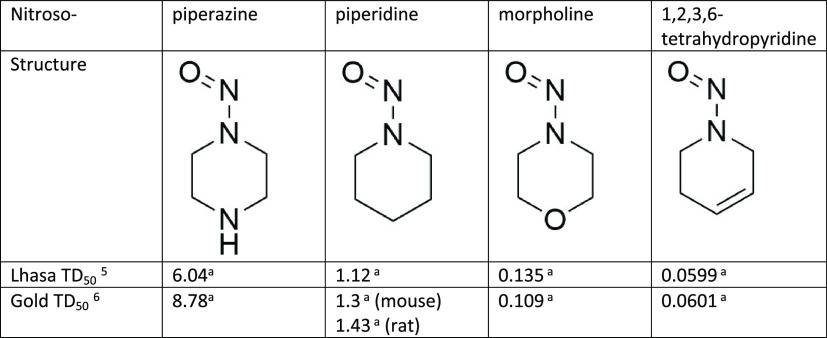
Potency Difference between Common Six-Membered Ring Systems

a All TD_50_ values are summary TD_50_ in mg/kg/day in rats unless specified, taken from the LCDB.^17^

### Naïve Feature Selection

A naive approach to identifying significant features is to compare the number of carcinogenic compounds with the feature to the number without the feature. This is analogous to a classic cross-sectional study, where a contingency table is generated and the probability of the feature influencing the carcinogenicity can be calculated using Fisher’s exact test.^22^ To test for potency rather than classification, a similar approach can be applied where the set of compounds are split by the presence of a feature and a t-test is performed on the log-potencies.

Performing Fisher’s exact test^22^ to compare the prevalence of carcinogenic compounds with a feature compared to those without the feature for the 25 features for which there is classification data available with a standard threshold of *p* < 0.05 results in four significant features (see Table 2) most of which make compounds less likely to be carcinogenic than the dataset as a whole. However, this method is hindered by the comparison of multiple features. After applying the Bonferroni correction to account for multiple testing (performing simultaneous statistical tests), only the presence of a carboxylic acid group anywhere in the molecule is found to have a significant impact at *p* < 0.0020. The Bonferroni correction (dividing the ideal *p*-value threshold by the number of concurrent statistical tests) is necessary, since when multiple independent tests (such as this *n*-fold classification exercise) are performed on the same dataset, the probability threshold required to reject all null hypotheses must be lowered. A simple example of this is the case of two concurrent tests, each significant at *p* = 0.05. The probability that at least one null hypothesis is nevertheless true is therefore 1 – (1 – 0.05)^2^ = 0.0975—thus even with two tests, considering significance for each at *p* = 0.05 results in significance for the family of tests of *p* = 0.1. In the case of this model, with 25 concurrent tests, the probability of at least one error (errors in this case are incorrectly rejected null hypotheses, i.e., false positives, features incorrectly considered significant) if a threshold of 0.05 were taken for each test is thus 1 – (1 – 0.05)^25^ = 0.723, i.e., 72%! Using the Bonferroni correction, this number is returned to ∼0.05. Of the 23 features which are represented in compounds where TD_50_ data is available, no feature is associated with significantly higher or lower potency (Table 3) using a *t*-test on log(TD_50_) at the corrected confidence of *p* < 0.0022.

**Table 2:** Significance of Different Features for Prevalence, According to the Naïve Feature Selection Method

feature	support	direction^a^	*p*-value	Bonferroni-corrected*p*-value	significant after Bonferroni correction^b^
carboxylic acid anywhere	13	less positive	0.000609	0.015231	yes
has *tert*-butyl	4	less positive	0.003573	0.089327	no
has isopropyl	24	less positive	0.019785	0.494619	no
has Et/Me	50	more positive	0.032018	0.800460	no
has α-CH_2_	7	more positive	0.067631	1.000000	no
has strong β-EWG	3	less positive	0.156232	1.000000	no

a The direction column denotes whether the presence of the feature was associated with more or less likely to be potent compounds than its absence.

b After applying the Bonferroni correction to account for multiple features tested, the significance threshold is 0.002 to provide an equivalent confidence to the *p* < 0.05 threshold for a single test.

**Table 3:** Significance of Different Features for Potency, According to the Naïve Feature Selection Method

feature	support	direction^a^	*p*-value	Bonferroni-corrected*p*-value	significant after Bonferroni correction^b^
Et/Me only	3	more potent	0.012498	0.287443	no
has isopropyl	6	less potent	0.018977	0.436477	no
piperidine	4	less potent	0.022392	0.515008	no
has weak β-EWG	5	more potent	0.053022	1.000000	no
has α-CH_2_	2	more potent	0.098168	1.000000	no
ring size 6	12	less potent	0.145844	1.000000	no

a The direction column denotes whether the presence of the feature was associated with more or less potent compounds than its absence.

b After applying the Bonferroni correction to account for multiple features tested, the significance threshold is 0.0022 to provide an equivalent confidence to the *p* < 0.05 threshold for a single test.

Treating the features independently fails to account for the presence of confounding features; for example, using the classification data there are 13 compounds with carboxylic acid groups anywhere (significant at *p* = 0.0006), two of which also have ethyl or methyl groups (significant at *p* = 0.03) and four of which have isopropyl groups (significant at *p* = 0.02). Similarly, using the regression data there are six compounds with the isopropyl groups (significant at *p* = 0.02) of which three also are substituted piperidines (significant at *p* = 0.02); as there are only four piperidine compounds in the dataset, this makes up 75% of the piperidine compounds.

As a result, one cannot say with any certainty whether the decrease in potency observed in these 25 features is real, a false positive caused by multiple testing—i.e., a statistical artifact—or whether an observed change in potency is due to confounding features rather than the feature of interest. Alternative, and more complex, modeling methods are thus required to handle this multiple-testing problem and allow true evaluation of the impact of different features.

### Bayesian Model Specification

To specify a minimal model of potency impact, four assumptions were made as follows:

(1) The distribution of nitrosamine potencies is log-normal.
(2) The presence of a feature will have some multiplicative effect on a compound’s potency (e.g., it will halve or double the TD_50_).
(3) The impact of multiple features on a single compound is independent.
(4) Features are more likely to have a smaller impact on potency than a larger one.

We have previously shown^15^ that the distribution of TD_50_ values for nitrosamines with known carcinogenicity data strongly matches a log-normal distribution. For simplicity, it can be assumed that the presence of a given feature will affect the potency of a compound in a consistent manner and that this is independent of the rest of the compound. While this does assume independence of feature effects, it does not require this independence in the dataset and so this assumption does not lead to the same problems as the naïve method discussed previously. Crucially the Bayesian prior acts as a regularizing term; this means that the multiple-testing problem is averted,^23^ and there is no need to apply the Bonferroni correction when evaluating statistical significance. The change in potency could either be treated as a constant absolute value (e.g., the feature increases the TD_50_ by 10 mg/kg/day), or a constant factor (the feature doubles the TD_50_). If in general the properties affecting a compound’s potency influence the TD_50_ as multiplicative factors this would imply that the effects on log(TD_50_) are additive. The central limit theorem, which states that under broad assumptions the sum of independent variables converges on a normal distribution, would then suggest that the distribution of the log(TD_50_)s resulting from the sum of the feature effects is normal, and so that the observed potencies are log-normally distributed. In this view, all variables that influence a compound’s potency do so in a multiplicative manner, and the features used by the model are a subset of these.

While multiple features may have synergistic effects, given the limited data available it is not possible to account for the large number of possible synergies. Taking only pairwise synergies of the 23 regression features would result in 23^2^ or 529 effects to be estimated from less than 70 compounds giving a severely underdetermined system. As it is not possible to account for these effects in a reliable manner, and that independent effects plus the central limit theorem provides a parsimonious explanation for the overall distribution, any synergistic effects are assumed to be negligible, and thus features can be considered to be independent.

While the effect of a specific feature is not known *a priori*, it is expected that most features will make no or little difference to the potency; however, it cannot be ruled out that some features may have large effects, possibly causing changes in potency (either increases or decreases) of many orders of magnitude. These two properties suggest a zero-centered heavy-tailed distribution whose domain covers both positive and negative values as an appropriate choice of prior distribution for effect sizes.

**Figure 3:**
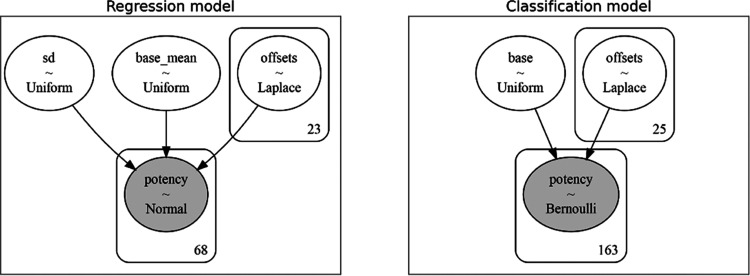
Specifications for the regression (left) and classification (right) models.

To create the posterior distribution, let μ and σ be the mean and standard deviation, respectively, of some normal distribution representing the potency of a hypothetical nitrosamine-containing compound with no features. Given the assumptions above the expected distribution of a given compound c can be defined as
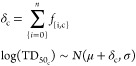
where *f*_i,c_ is the effect of feature i on the expected potency of compound c. This can be formulated as a linear regression problem with the addition of a prior distribution over *f*. While any distribution matching the criteria specified above would be a suitable prior, a Laplace distribution^24^ was used since the “peakedness” of the Laplace distribution is consistent with the idea that most features will have no effect, while the heavy tails allow sufficient freedom for parameter estimates for those few features that do have significant effects. Values of the 50 and 95% intervals were also in line with expert intuition. Minimally informative uniform priors were used for μ, and σ giving the model shown in Figure 3. While the resulting model is a regression model, unlike with ordinary linear regression, we are interested in inferring the parameter values and associated uncertainty of the regression coefficients rather than the predicted potencies themselves, as such its use is analogous to a statistical test rather than a classical regression model. For the classification problem, a similar technique can be used with the observations being Bernoulli trials^25^ with probability
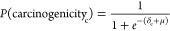


Both the regression and classification models were implemented in python (version 3.7) using pymc3.^26^ Inference was performed using Markov chain Monte Carlo^26^ methods. Models were run separately for both the nitrosamine and NOC datasets.

### Comparison with Expert Knowledge

A truly blinded comparison proved impossible to recreate since, given the current status of the nitrosamine crisis, anyone with sufficient knowledge of nitrosamine chemistry to make predictions is aware of the more potent carcinogens. However, one of the authors, expert in nitrosamine mutagenicity classification SAR, was provided with a list of the features but not access to the carcinogenicity data and asked to classify features as to whether they would be expected to increase or decrease carcinogenic potency to provide an expert assessment for comparison.

## Results

Both the regression and classifications models were run using four chains of length 10 000. No divergences were found during sampling, and Gelman–Rubin values^27^ of less than 1.001 were seen for all parameters indicating the models converged on a stable solution. The expected baseline potency for a hypothetical nitrosamine with none of the selected features was estimated at 1.9 mg/kg/day, with estimates ranging from 0.3 to 12.5 mg/kg/day (mean ± std of log-potency) due to uncertainty in the effects of the features and the limited data available. For comparison, a naïve estimate of the baseline potency given by the geometric mean of the TD_50_s is 0.86 mg/kg/day, approximately half the model estimate but well within the uncertainty range given. This suggests that the features selected are causing a net increase in potency using the model estimates. The potential impact of the “featureless nitrosamine” concept will be discussed subsequently.

The expected baseline probability of a hypothetical nitrosamine with no selected features being carcinogenic was estimated at 78%, with estimates ranging from 56 to 90% (mean ± std of log-odds-ratio) due to uncertainty in the effects of the features and the limited data available. For comparison, a naïve estimate of the baseline prevalence based only on the number of positive calls puts the baseline probability at 75%—very close to that estimated by the model.

For both regression and classification modeling, a *k* of 1 (the sole hyperparameter required) was used for the prior Laplace distribution following a search over a range of *k* values. In the regression model, this corresponds to a 50% confidence of an effect size of a less than 4.9-fold change in potency and a 95% confidence of a less than 990-fold change; for classification, this equates to a 2-fold and 20-fold change in probability at 50 and 95% confidence, respectively.

The regression model was found to be insensitive to variations in the prior with the magnitude of effect being consistent for most features over the range tested. Notable exceptions are N_4_-substituted and unsubstituted piperazines, those compounds where both carbons are aromatic, and those containing an isopropyl group or benzylic group. With the exception of benzylic groups, which increase potency, these features were predicted to decrease potency over all priors; however, the magnitude of the change increases as the priors are relaxed. For all features including the four previously mentioned, the confidence of an effect, i.e., the point in the sample distribution where it is crossed by the line of no effect, was consistent across the range of priors tested. Leave-one-out cross-validation was used to estimate the goodness of fit for each prior with the result that the tighter, more informative priors performed better. In this situation, outside knowledge must be balanced against the goodness of fit to arrive at a suitable prior. Given the similarity in results between the models and strong evidence of the importance of structural features on potency, the wider prior of *k* = 1 was retained.

The classification model was found to be much more sensitive to the choice of prior, with the magnitude of the effects varying as the prior is relaxed. This is likely due to the decreased information contained in a binary positive/negative call rather than a potency value—while more compounds are available, the total information going into the classification model is less than the regression model. Like the regression model, the confidence of a feature having some nonzero effect is more stable across prior estimates—especially for features which are predicted to have no impact on carcinogenicity. For more details, see the Supporting Information.

### Regression

**Figure 4:**
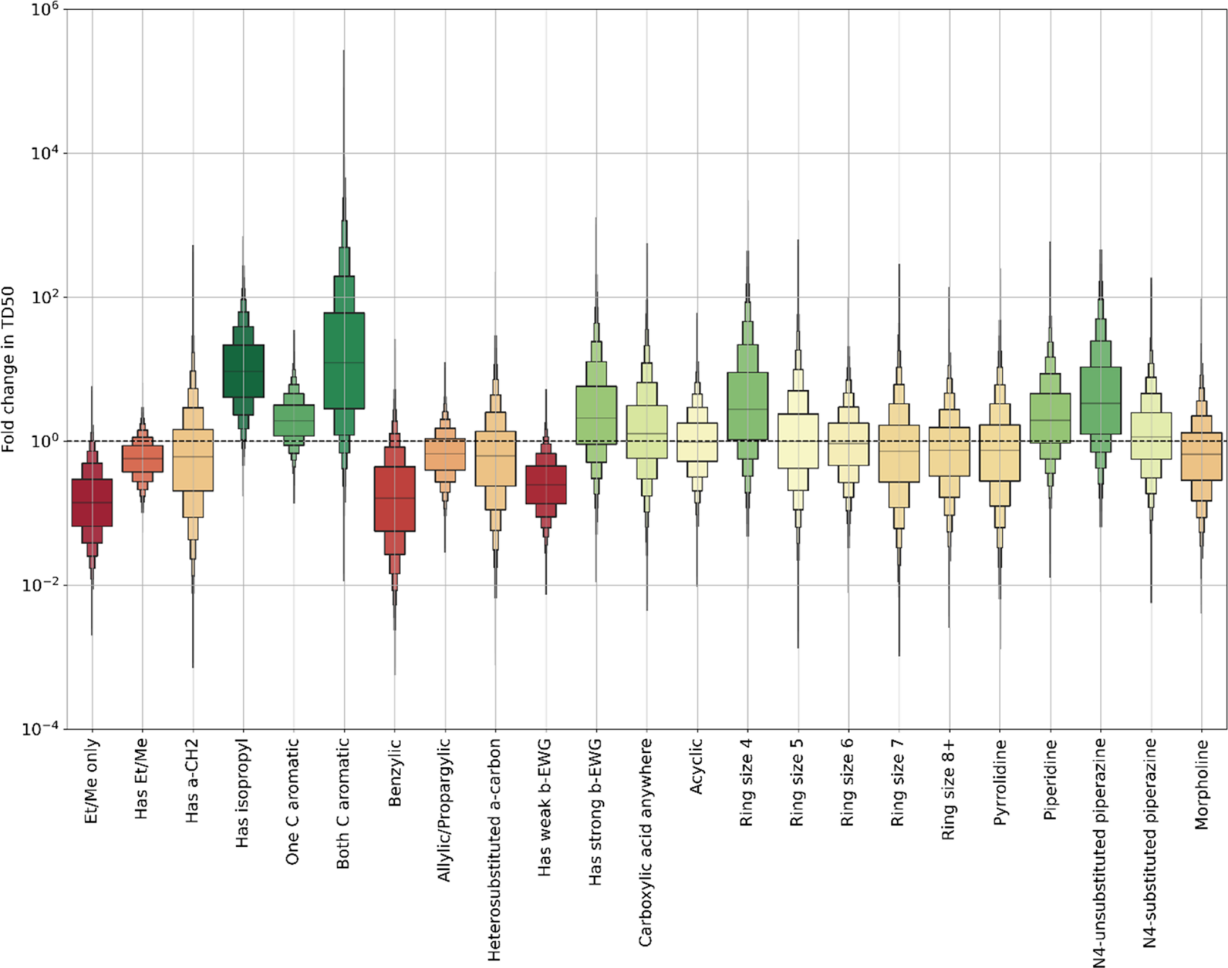
Statistical impact of features on nitrosamine carcinogenic potency. Boxes indicate median and a series of quantiles of the predicted change in TD_50_ value caused by presence of a given feature.

Figure 4 shows the predictions made by the potency model, and Table 4 shows selected *p*-values (cf. Table 3 for the naïve model). It is seen from Table 4 that some features that were thought to be of significance in the naïve model no longer are. As discussed previously for piperidines and six-membered rings in general, many of the molecules containing these features have been evaluated to investigate the effect of features such as steric hindrance, and—while this feature overlap should not distract an expert analysis, statistical models that fail to account for this would assign undue importance to these features. It is particularly worth noting that no significance is associated with the presence of an α-CH_2_ group; this is presumably because the vast majority of compounds for which we have data have this feature, and those few that do not probably match other features, having by definition two aromatic, isopropyl, or *tert*-butyl substituents, and the statistical effects are better associated with those other features.

**Table 4:** Significance of Selected Features for Potency, According to the Bayesian Multiple Linear Regression Model Described

feature	direction^a^	*p*-value	significant
has isopropyl	less potent	0.0283	yes
Et/Me only	more potent	0.0326	yes
has weak β-EWG	more potent	0.0510	clear trend but not formally significant
benzylic	more potent	0.0954	clear trend but not formally significant
both C aromatic	less potent	0.101	clear trend but not formally significant
The following were among the lowest *p*-values in the naïve model, as listed in Table 3, but no longer are. Their *p*-values from the Bayesian model are given for comparison.			
piperidine	less potent	0.273	no
has α-CH_2_	more potent	0.349	no
ring size 6	less potent	0.462	no

a The direction column denotes whether the presence of the feature was associated with more or less potent compounds than its absence.

With the Bayesian multiple linear regression model, three features show an association with a large increase in potency with respect to the hypothetical featureless nitrosamine, i.e., are associated with greater potency. Note that, due to the size of the dataset, we discuss here some features that are not formally statistically significant, but would be expected to be so were a larger dataset available. These are:

(1) Nitrosamines with only ethyl or methyl groups. A closed set of three compounds, known to be highly potent, and arguably the archetypical nitrosamines. This potency is well established,^28^ and it is of more relevance to establish the effect of considering the rest of the dataset in the absence of these three (which may well be a justifiable assumption, given the differences between this closed set and larger nitrosamines^14^) than to re-state previous discussions of their activity.
(2) Benzylic nitrosamines. This larger set of compounds (defined more broadly than simply phenyl–CH_2_–NN=O to include all aromatic systems), while somewhat sterically hindered than simple nitrosamines, includes a feature that may be associated with increased potency since it is known^29^ that the benzylic position is particularly reactive due to conjugation with the aromatic system and thus offers enhanced metabolism. It is also worth commenting that the benzylic nitrosamines in the dataset are often tobacco-specific nitrosamines (TSNAs), such as NNN^30,31^ and analogues, and have thus been studied to an unusually high degree.^32,33^
(3) Compounds with weak^34^ β-position electron-withdrawing groups (EWGs). This may at first seem contradictory to the observation in Cross and Ponting^14^ that compounds with strong β-position electron-withdrawing groups are negative; however, the majority of the *weak* electron-withdrawing groups in the dataset are ketones. While these are undeniably electron-withdrawing, the α-hydroxylation of the nitrosamine is also α-hydroxylation of a ketone, a process known to be metabolically favored due to conjugation with the ketone, which results in an acidic, and thus easier to remove, α-hydrogen. Furthermore, 2-oxopropyl groups have been observed to lead to an alternate methyl adduct via an intramolecular rearrangement following α-hydroxylation on the other side of the nitrosamine; the same may apply to 2-oxobutyl and larger.^35^

Two features show an association with a large decrease in potency with respect to the class averages:

(1) Those compounds with at least one isopropyl-like group (i.e., the α carbon has two carbon substituents). The presence of even one isopropyl group leading to a reduction in potency may be an extension of the observation that a *tert*-butyl group leads to an elimination of the potency and the reasons for it—while less sterically hindered than a *tert*-butyl, the isopropyl is less likely to be a site of metabolism than a CH_2_ group and, should metabolism occur on the other side of the nitrosamine, the formed diazonium or cation will be less reactive with DNA than a CH_2_ group. A comparison can be made between nitrosopiperidine (summary TD_50_ of 1.43 mg/kg/day in rat) and 2-methyl nitrosopiperidine (summary TD_50_ of 13.2 or 20.4 mg/kg/day, depending on enantiomer^36^—the difference between these latter two values may be within experimental variation), and has been made subjectively by Lijinsky and Taylor.^37^ No category has been made for compounds with two isopropyl groups since this can be described as a linear combination of other features and thus reduces model independence; however, the presence of two isopropyl groups should be associated with a further reduction in potency. This is borne out in the data; NDIPA (nitrosodiisopropylamine) is a subjectively weak carcinogen,^12^ though no TD_50_ has been reported, and cyclic analogues such as 2,6-dimethyl nitrosopiperidine are reported to be negative in the aforementioned study.^37^
(2) Those compounds where both carbons are aromatic. These compounds are *a priori* unable to undergo α-hydroxylation due to containing no α-hydrogen, and the example that is in the dataset is the compound with the single weakest potency that remains positive (nitrosodiphenylamine, (NDPhA)^38^); however, since it is only a single example, the statistical power of this observation is limited and the confidence interval broad. An alternative mechanism of action to α-hydroxylation must be proposed here.

### Classification

**Figure 5:**
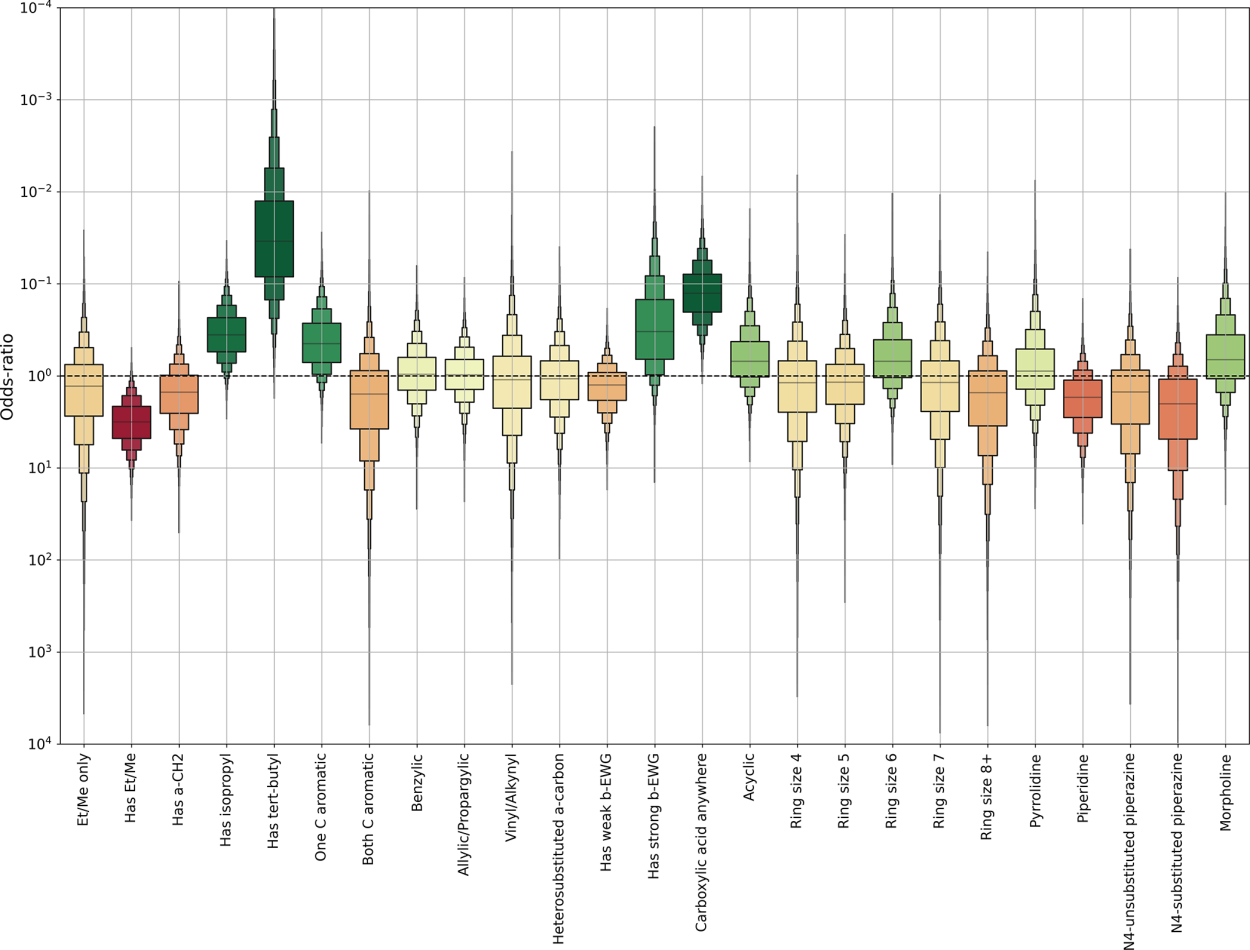
Statistical impact of features on nitrosamine carcinogenic positive prevalence, expressed as an odds ratio. Boxes indicate median and a series of quantiles of the predicted change in prevalence caused by the presence of a given feature. A value of 0.1 is 10 times less likely to be carcinogenic, likewise a value of 10 is 10 times more likely to be carcinogenic.

Figure 5 shows the predictions made by the Bayesian classification model, and Table 5 shows selected *p*-values (cf. Table 2 for the naïve model). One feature that was considered of potential interest in the naïve model, the presence of an α-CH_2_ group, was not of particular importance in the Bayesian model. Comparable to the case for the same feature in the regression potency model, the impact of this feature on prevalence is captured first by the features that describe its absence—isopropyl, aromatic, and *tert*-butyl side chains, and also by the ethyl or methyl groups that *a priori* also match this feature.

**Table 5:** Significance of Selected Features for Prevalence, According to the Bayesian Classification Model

feature	direction^a^	*p*-value	significant
carboxylic acid anywhere	less positive	0.000925	yes
has *tert*-butyl	less positive	0.00178	yes
has Et/Me	more positive	0.0150	yes
has isopropyl	less positive	0.0428	yes
one C aromatic	less positive	0.108	clear trend but not formally significant
has strong β-EWG	less positive	0.115	clear trend but not formally significant
The following were among the lowest *p*-values in the naïve model, as listed in Table 2, but no longer are. Their *p*-values from the Bayesian model are given for comparison.			
has α-CH_2_	more positive	0.256	no

a The direction column denotes whether the presence of the feature was associated with more or less potent compounds than its absence.

Only one feature was associated with a significant increase in positive prevalence with respect to the class prevalence (78%, as discussed)—those compounds with ethyl or methyl side chains. It could conservatively be assumed, therefore, that all dialkyl nitrosamines with ethyl or methyl side chains should be considered potentially carcinogenic.

Three features show a significant decrease in positivity; these are, in order of effect size: compounds with *tert*-butyl groups, compounds with carboxylic acids anywhere, and compounds with isopropyl groups. Two more features show strong, but not significant at *p* = 0.05, effects; these are those compounds with strong β-electron-withdrawing groups or those where one of the carbon substituents is aromatic.

(1) *Tert*-butyl groups: The effect of these on classification is strong enough that this feature does not occur in the graphs for regression—there are no nitrosamines with *tert*-butyl groups that have been reported to be positive. The reasons for this have previously been discussed.^12−14,39^
(2) Compounds with carboxylic acids anywhere: These compounds are typically negative for a different reason to local effects around the nitrosamine; rather, the presence of the acid makes affects the physicochemistry and pharmacokinetics of the molecule as a whole. First, carboxylic acid-containing molecules are typically strongly bound to plasma protein,^40−44^ which may reduce the peak exposure and thus the potential for a sufficient rate of mutagenesis to overwhelm repair and ultimately induce tumor formation. Second, the compound is much more hydrophilic such that the opportunity for it to be α-hydroxylated is dramatically reduced and elimination without the need for phase I metabolism becomes plausible.^11,45,46^ This combination of increased plasma-protein binding and enhanced clearance is known to significantly reduce the bioavailability and thus efficacy of drugs;^41,44^ this effect can be extrapolated to nitrosamine toxicity to explain the reduced prevalence and potency *in vivo*. This is also a useful place to discuss the interplay of different features: Nitrosomethylbutanoic acid (NMBA) is a moderately potent bladder carcinogen—not hepatic—despite having a carboxylic acid;^47^ however, it does also contain a methyl group, which, as has been discussed, can be assumed to indicate a positive result. While no data exists, these observations should be able to be extrapolated to bioisosteres of carboxylic acids.
(3) Compounds with isopropyl groups: The association of these with negative results may be due to the increased steric hindrance of the isopropyl group, especially in the cases of those compounds with two isopropyl groups—which, when cyclic, appear to be especially associated with negative results.^12−14^
(4) Compounds with strong^34^ β-EWGs: As has previously been noted,^14^ these are associated with a decrease in potency, and where both sides of the nitrosamine have strong β-EWGs these are negative—which, given the prior assumption of positivity, gives a substantial change in the odds ratio toward negativity even though the four compounds which match this feature are evenly split. One reason for this may be due to the strong EWG reducing the availability of the α-hydrogen for metabolic hydroxylation, strengthening the C–H bond as electron density is withdrawn from the carbon, which forces the formal transition state to a more product-like, harder to achieve, conformation. A potential alternate hypothesis is that the EWG impacts the rate of subsequent steps, changing the relative rates of DNA alkylation and detoxification via reaction with water; while quantum-mechanical calculations outside the scope of this manuscript would be required to confirm whether the effect on the metabolic activation or DNA reaction are more important, the decreased potency of *N*-2,2,2-trifluoroethyl-*N*-nitroso ethylamine with respect to NDEA (which has a free ethyl group available for facile metabolism) suggests that there is some impact. The EWG must however be strong, such as CF_3_ and C≡N. The definition of a strong EWG has previously been simplified^14^ from the extensive list of Δ*V*_c_ values provided by Remya and Suresh^34^ to those most commonly found in pharmaceutically relevant molecules; but critically excludes those with weak EWGs such as ketones that are, as discussed (Figure 4), associated with increased potency. While the impact of the C–H bond strength would reduce the potency for the ketone-derived EWGs, the increased acidity via the enol tautomer, and presence of the rearrangement mechanism^35^ discussed, counteract this.
(5) Compounds with one aromatic substituent: The presence of the aromatic substituent prevents α-hydroxylation at that side, requiring either metabolic oxidation to occur at the other side—which may or may not be possible, hence the reduction in positivity—or an alternative mechanism to α-hydroxylation to occur (which is the case with the one exemplar where both carbons are aromatic—NDPhA is positive, but an exceptionally weak carcinogen,^38^ potentially via transnitrosation^48,49^ to the aryl nitroso analogue;^50^ since it is a single positive example, it is not statistically significant for classification but has previously been discussed). Where they are carcinogenic, the ultimate reaction with DNA differs from aliphatic amines, in that nucleophilic substitution of the diazonium does not occur, aromatic carbons not being suitable substrates for SN1 or SN2; rather the initial DNA adduct formed retains the diazo group (Ar–N=N–DNA).^51^ As discussed subsequently, there appears to be a trend where potency may be correlated with the substitution pattern on the aromatic ring and thus with the electronic interactions of that ring with the diazonium ion or other mechanistic intermediates such as the diazohydroxide. For many compounds in this class, the electron-withdrawing nature of the ring is sufficient to move them from carcinogenic to noncarcinogenic.

### NOC Dataset

All nitroso compounds (the NOC dataset) were treated similarly. The results did not differ much for those features which are found both in the dialkyl nitrosamine compounds. However, moving to the larger chemical space of the different classes of NOC, it can be noted that nitrosated hydroxylamines or alkoxylamines are associated with significantly reduced potency and *N*-nitrosocarbamates with increased potency with respect to the hypothetical featureless NOC, and in classification terms, nitrosoureas are more likely to be positive than the featureless NOC.

The observation of significantly reduced potency for heteroatom-substituted nitrogens, yet retained potency for *N*-nitrosoamides and similar compounds allows some boundaries to be set to the scope of the cohort of concern. In particular, it appears that the *N*-nitroso group must be substituted with two carbon atoms (heteroatoms lead to low potency, and nitrosated primary amines are unstable^52^), and if these are alkyl at least one α-hydrogen is required.

*N*-Nitrosoamides and related compounds are expected to have similar SAR with respect to DNA alkylation as nitrosamines—the DNA-reactive species is still a diazonium ion—but do not require metabolic activation;^32^ thus, a different overall SAR would be expected, though comparable trends have been observed (Me > Et > allyl > Pr > Bu) based on the reaction of nitrosoureas with trapping agents.^53^

The inclusion of additional types of NOC also allows the “nitrosamine” feature itself (i.e., all nitrosated secondary amines) to be analyzed. While the effect size is small, nitrosamines are slightly *less* likely to be positive than the median NOC (driven by the strong positive prevalence of *N*-nitrosoureas) but, where positive, fractionally *more* potent. These effects are not statistically significant. Full figures comparable to Figures 4 and 5 are in the Supporting Information.

## Discussion

### “Featureless” Nitrosamines

As previously introduced, the methods used here allow the investigation of a hypothetical “featureless” nitrosamine. While chemically impractical—the set of features used cover chemical space almost entirely, excepting only nitrosated ammonia (H_2_NN=O, H_3_N^+^N=O) and nitrosated tertiary compounds (i.e., R_3_N^+^N=O, R anything except H)—this hypothetical is a useful reference point. Both activating features such as ethyl/methyl groups, and deactivating features such as *tert*-butyl groups, are removed from the possible chemical space of the featureless nitrosamine. This hypothetical nitrosamine has a 78% chance of being carcinogenic, reflecting the distribution as a whole, and an expected TD_50_ if positive of 1.9 mg/kg/day, corresponding to an AI of 1.9 μg/day. This is significantly higher than the current regulatory limit set by the EMA^1^ of 18 ng/day and reflects the differences between the potent, small-molecule nitrosamines^14^ that lead to the setting of such limits—either explicitly, in the case of compounds read across to, e.g., NDEA, or implicitly, in the case of the compounds that define the 5th percentile,^15^ used by the EMA and others, by virtue of being the most potent.

It is also important to stress that, where a class is observed to be “more potent” or “more likely to be positive”, in this article this is with respect to this featureless nitrosamine (predicted TD_50_ 1.9 mg/kg/day, 78% chance of being positive) rather than to the archetypical NDEA/NDMA.

The authors do not by any means recommend the increase of the class-specific limit to that of the featureless nitrosamine, since nitrosamines do have structural features; however, the absence of potency-increasing, or presence of potency-reducing, features has been shown in this work to lead to statistically significant differences between NDEA and similar compounds and the hypothetical featureless nitrosamine. Chemical reasons for these differences have recently been explored.^14^ This implies that the absence of certain features, or presence of others, could be considered sufficient information to increase the AI for a compound to a value that is significantly above the class-specific limit. Several methods have been proposed for how the AI for the compound should then be set: Dobo et al.^51^ have demonstrated that this could be done by taking a defined structural class which contains the compound and taking the lowest reliable TD_50_ value in that class (though that does lead to conservative values, since, e.g., in the case of the pyrrolidines, the lowest reliable TD_50_ is that of NNN with its activating benzylic group), Our previous work showed that this could be performed using the distribution to estimate a 5th percentile for the class,^15^ and in this work, we propose a method using the statistically significant features to assign compounds into order-of-magnitude-based brackets, as presented below. The set of classes used for an approach like this should sufficiently cover chemical space in a manner comparable to the classes in this work or those in Dobo et al.,^51^ and the significance of each feature should be analyzed using this or similar methods. Expert analysis is required where a compound is in multiple classes, although as noted below those compounds with features that increase and decrease potency are typically of medium potency. When designing sets of features it should be taken into account that both the presence and absence of a feature contain useful, and complementary, information: for example, there is no need to include both sterically hindered (i.e., the considered, but not included “no α-CH_2_” feature) and not sterically hindered (the included “has α-CH_2_”) as features. Indeed, as these cannot be considered independent features, doing so would violate the model assumptions.

### Evaluation of Key Features

#### Ethyl/Methyl Groups

Comparing the two models, i.e., regression modeling on the potency data and classification modeling on the overall carcinogenicity result, leads to some surprising observations—some results from the two models appear contradictory. The most obvious case for these is comparing those compounds with only ethyl/methyl groups with the set of those that have at least one ethyl/methyl group; the former is significant for potency but not for positivity, and the latter for positivity but not potency. The reasons for this difference stem both from the inherently biased nature of the dataset and the nature of these two classes. It will be seen from Figure 4 that those compounds with only ethyl or methyl groups show a strong trend toward potency—driven by the known extreme potency of the three examples—but are not significant in classification terms. This is because there are only three examples, which is not enough to provide a statistically significant trend toward positivity, especially in the context of the hypothetical featureless nitrosamine being 78% likely to be positive. By contrast, the presence of ethyl or methyl groups in a larger molecule does not provide a statistically significant increase in potency. This is due presumably to the changed nature of the alkylating diazonium ion that is formed from the other side of the molecule, or alternative metabolic or pharmacokinetic fates available to a larger molecule. These groups do however have a strong association with positivity—and with many more than three examples, this is sufficient to be significant which was not the case for those with only ethyl or methyl groups. To summarize, the following conclusions can be drawn:

- NDEA, NMEA, and NDMA are of unusually high potency (even by comparison with other nitrosamines)—and all three are known to be carcinogenic.
- All compounds with ethyl/methyl groups are very likely to be carcinogenic (even by comparison with other nitrosamines).

#### Steric Hindrance

The next categories that lead to unexpected differences between the models are those compounds with strong steric hindrance—aromatic, *tert*-butyl, and isopropyl groups. In general, the presence of one or two of these groups leads to a significant reduction in both positivity and potency; however, the category for compounds with two aromatic groups is *more* likely to be positive. This is an example of limitations of statistical power due to dataset size—data is only available for one compound in this class (NDPhA^38,54^) and it is positive, but of extremely low potency, resulting in a mismatch. The *tert*-butyl groups, on the other hand, give a sufficient reduction in prevalence that the category does not exist in Figure 4—we have no positive examples from which to calculate potency data! Contrary to this, the presence of one aromatic group (which by definition lacks at least one α-hydrogen) does not remove prevalence or potency, though it does decrease it- this may be due to the potential alternative mechanism of DNA alkylation for this class which does not depend on S_N_1/S_N_2-mediated cleavage of the C–N bond.^51^ A sufficient increase in the degree of steric hindrance to remove all α-hydrogens has been recognized as reducing or eliminating carcinogenic potential;^1^ however, this is due to the α-hydroxylation mechanism (i.e., the mechanism that leads to a cohort of concern-level potency) becoming impossible rather than the hindrance itself. For these categories, the following conclusions can be drawn:

- The presence of sterically bulky groups (i.e., any degree of substitution on the α-carbons) leads to a reduction in potency and prevalence.
- This effect is magnified as the degree of steric hindrance increases.
  - The presence of even one *tert*-butyl group leads to negative results.
  - Having two aromatic groups leads to extremely low potency—and although not a negative result, these compounds can be argued to be outside the cohort of concern.

#### Electronic Conjugation

Considering substitution patterns further, several rules can be drawn. Benzylic—including all compounds that match the substructure aryl–C–NN=O—nitrosamine compounds (and by extension potentially allylic and propargylic, though with a significantly smaller effect size, and there is no data available for propargylic nitrosamines but due to known similarities in reactivity between allyl and propargyl systems the behavior of allylic can be extrapolated to propargylic) and compounds with weak^34^ β-EWGs, which, while defined more broadly in the patterns are effectively restricted to carbonyl compounds in the available dataset, are associated with an increase in potency but have no effect on prevalence. This indicates that, if positive, these compounds are likely to be potent carcinogens. This may be due to the metabolic “hot-spot” represented by the benzylic/allylic/propargylic carbon—it is notable that allylic and benzylic C–H bonds have a bond dissociation energy (BDE) over 10 kcal mol^–1^ lower than alkyl sites,^55^ which indicates a much higher reactivity toward C–H activation such as metabolic α-hydroxylation—or the acidic α-carbon of the ketone (which is of course also the α-carbon of the nitrosamine). For the β-oxoalkyl compounds, an alternative, rearrangement, mechanism is available which results in a methyldiazonium ion should the other side of the molecule be a suitable substrate for metabolic activation.^35^ This methyldiazonium is the same DNA-alkylating agent as in the case of NDMA and may be an additional reason for the increased potency. The benzylic position is metabolized even in preference to the methyl in *N*-methyl-*N*-nitrosobenzylamine^56^ and is at least as available to metabolic oxidation via some P450 isozymes as the methylene CH_2_ position in Nitrosonornicotine (NNN),^31,57,58^ despite steric hindrance (this site in NNN falls into the isopropyl group category). The other benzylic compounds in the potency dataset are analogues of NNN (Figure 8b); of these, the NNN *N*-oxide is also more potent than nitrosopyrrolidine, though less potent than NNN, whereas the nitrosoanabasine is, surprisingly, of lower potency than nitrosopiperidine—potentially due to a significantly increased potential for a competitive detoxifying (via introduction of a hydrophilic^59^*N*-oxide) hepatic *N*-oxidation.^30,60^ As an aside, this shows the importance of considering clearance alongside metabolic activation in the consideration of more complex nitrosamines. On the other hand, strong EWGs that do not lead to a particularly acidic α-carbon, such as CF_3_ (electron-withdrawing via hyperconjugation to the C–F bond, as opposed to favoring the formation of potential negative charge via delocalization), and the presence of a carboxylic acid anywhere in the molecule, which affects the compound’s *in vivo* fate significantly,^11,40^ both lead to a reduction in the probability of a compound being positive, but if positive, it is likely to be of comparable potency to the featureless nitrosamine.

- Compounds with a group that increases the metabolic liability of the α-carbon are of increased potency.
- Decreasing the metabolic liability of this carbon via strong EWG substitution leads to decreased positivity.
- Changing the DMPK profile such that there is lower requirement for phase I metabolism leads to decreased positivity.

#### Cyclic Systems

Cyclic nitrosamines can be analyzed in two principal ways: first, by consideration of ring size. Patterns have been made for rings of size 3–7, and a combined category for all rings of 8 or larger. No statistically significant trends are reported here. Consideration of the simple alicyclic series (nitroso-azetidine, -pyrrolidine, -piperidine, -hexamethyleneimine, and -heptamethyleneimine) has been observed to show an increase in potency with ring size, with rings of size 4–6 being of lower potency than larger ones (nitrosopyrrolidine being an exception to this trend according to Gold TD_50_^6^ values, but that is driven by a single-dose study and the Lhasa TD_50_^5^ is higher than for nitrosopiperidine, as expected), but this trend is not replicated in a statistically significant manner as the chemical space is increased to cover greater structural diversity. It should be noted that other homologous series, such as the symmetrical alkanes, also show comparable trends, but there may also be mechanistic explanations such as the size or binding affinity of the different ring sizes.^58^ Figure 5 in Cross and Ponting^14^ would indicate that the data for chemical classes might support this, but upon closer investigation (not performed at that time), the following observations can be made: The four-, seven-, and eight-membered ring classes have a total of four examples between them, one of which has multiple nitroso groups, and the five-membered rings are of higher median potency. This higher median potency may be due to the presence in the dataset of a number of benzylic species, derivatives of NNN,^30−32^ whereas by comparison many of the six-membered rings for which data exists have been studied to investigate the effects of methylation and steric hindrance. This shows the power of the method described here, that a trend apparent from the raw data can be overturned when the effects of different features are isolated, overturning the sampling bias that would otherwise be derived from the small dataset.

Second, the impact of ring features can be considered. While not statistically significant, it is worth noting that the overall category for rings of size 6 has minimal impact, but breaking the six-membered rings down by sub-category indicates that (unlike trends with ring size) the trends observed for the nitrosopiperidine, morpholine, and piperazine mentioned in Table 1 carry over to the broader classes, with a significant caveat: N_4_-substituted nitrosopiperazines do not show the reduction in potency that is observed for the N_4_-unsubstituted, and their potency is closer to that of other six-membered rings. The decreased potency of the unsubstituted piperazines may be due either to the effect on pharmacokinetics of the secondary amine—potentially protonated *in vivo*—or allow for alternative detoxification pathways, which may require future quantum-mechanical investigation. Further effects may be due to the relative ratio of α- and β-hydroxylation in the different rings; where in nitrosopiperazines the two hydroxylation rates may be more similar due to increased similarity in chemical environment, in nitrosopiperidines and nitrosomorpholines the two positions are chemically more distinct. To summarize:

- Trends associating ring size with potency can be observed within homologous series; however, there is no *overall* association between size of ring and carcinogenic potency or prevalence.
- The structurally similar series of six-membered rings shows large potency differences based on the atom at the 4-position and the impact that has on pharmacokinetic and metabolic processes.

### Comparison of Models

**Figure 6:**
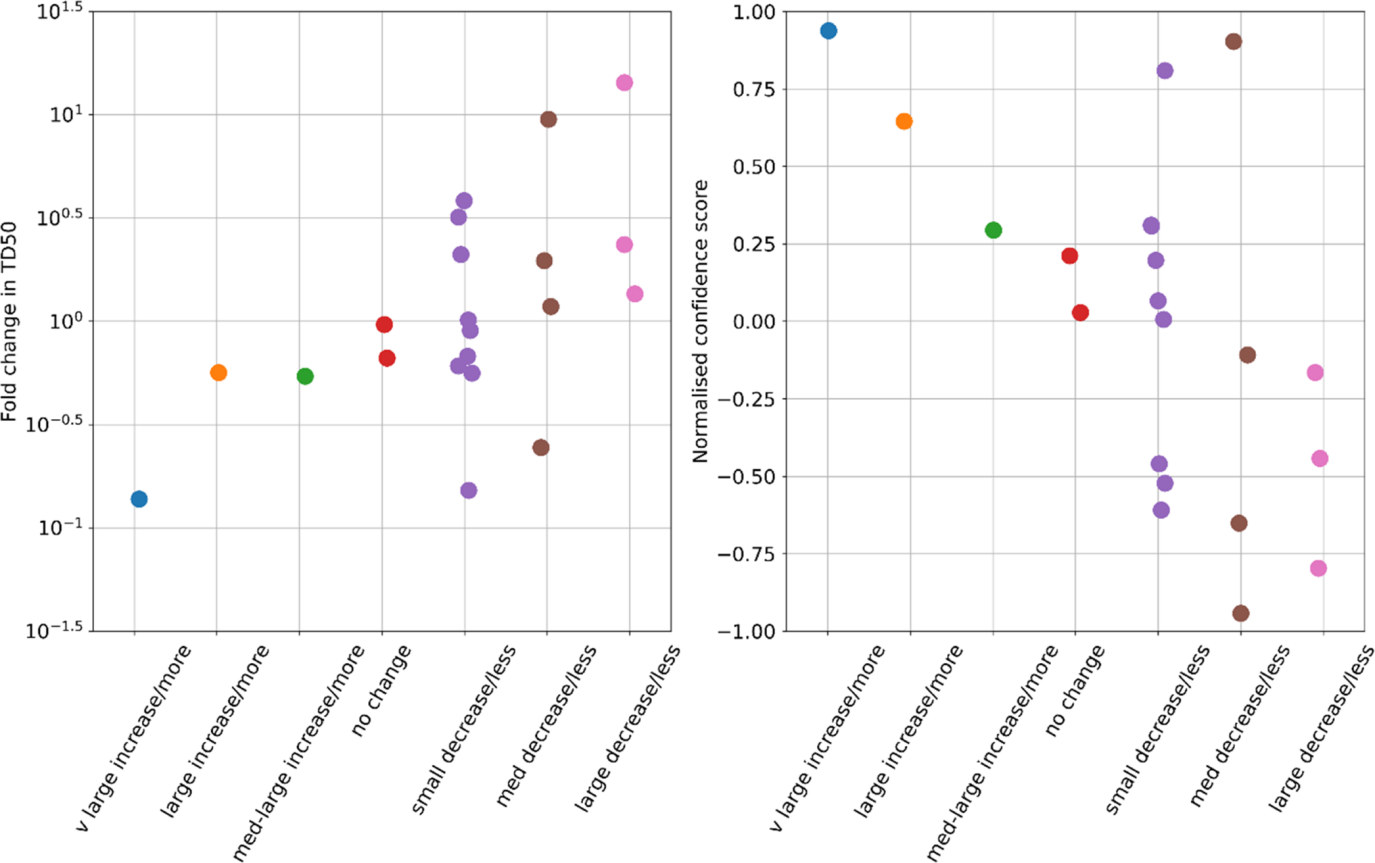
Comparison of model results with expert-derived predictions. (L) Predicted impact on potency. (R) predicted impact on positive prevalence. X-axes indicate expert assessment, Y-axes indicated model predictions for each feature.

*Post hoc* examination of the predictions, as has been performed above, can provide good explanations for the majority of the models’ predictions but can be prone to motivated reasoning, where justifications are found to “explain away” unexpected findings. It is therefore important to compare the model predictions against a set of blinded expert judgments. As the model presented here suffers from different limitations and biases to an expert, perfect agreement is not expected but a high level of agreement provides validation for both the expert opinion and the model predictions. As shown in Figure 6, there is strong agreement between the expert prediction (a correlation can be seen with the exception of two points—bottom right for potency, top right for prevalence—which are the features discussed below, though as features tend toward indicating lower potency/prevalence, the confidence margins spread), and both the magnitude of a predicted effect and the model’s confidence of an effect. In both cases, the expert predictions correlate well with the model predictions, with a Kendall tau^61^ of 0.47 (*p* < 0.01) for the predicted magnitude, and 0.46 (*p* < 0.01) for the model confidence.

There are however some notable differences between expert and model predictions. Of the 23 features being compared, only three were predicted to increase the potency by an expert; in comparison, 11 were predicted by the model. This is due to the differing baseline used by the model compared to the expert. The expert predictions are instinctively comparing potency relative to an already highly potent nitrosamine, such as NDMA or NDEA, whereas the model predictions are relative to the less potent featureless nitrosamine discussed previously. There are also two features where the expert and model predictions strongly disagree. Compounds with weak β-EWGs were predicted to have a moderate decrease in potency by the expert, but were predicted to increase the potency by the model (expected a 75% reduction in TD_50_, with a confidence of effect of 90%). Benzylic compounds were also predicted to give a small decrease in potency by the expert, whereas the model predicted an increase (expected an 85% reduction in TD_50_ with a confidence of effect of 80%). After further examination, these two features are typically associated with features that do offer significant decreases in potency (strong electron-withdrawing groups and steric hindrance, respectively), hence the expert assumption. The reasons that weak EWGs and benzylic groups do not give the assumed decrease in potency according to the model are, in both cases, that they promote rather than suppress the metabolic activity of the α-carbon; as previously described, the conjugation-induced increase in acidity outweighs the inductive decrease in metabolic liability. A full understanding of the metabolic profile of the nitrosamine is thus critical.

As discussed above, the predicted features impacting potency agree well with expert predictions allowing statistical weight to be given to expert assessments; however, the model does have a number of limitations. First, the assumption of independence of features places limits on what features, and combinations of features, can be assessed using this method. In reality, it is unlikely that features can be neatly divided into independently acting items, meaning some degree of dependence must be accepted. For example, the features “ethyl/methyl only” and “has ethyl/methyl group” cannot strictly be considered independent, the former being a subset of the later; however, the known importance of the “ethyl/methyl only” feature on potency makes it necessary to include. Including it as a separate feature also separates NDEA, NDMA, and NMEA from the larger group of ethyl/methyl-substituted nitrosamines with other features on the other side of the amine, allowing better evaluation of these. Additional synergistic effects may be present where a combination of two features has a greater impact than their individual effects. The clearest case where this may occur is the presence of steric hindrance due to an isopropyl, *tert*-butyl, or aromatic carbon. The presence of any one of these significantly reduces the potency and/or prevalence of carcinogenic activity, but the other side of the molecule may still be available for metabolic activation. Should both sides be hindered, metabolism at both sides is inhibited and potency and prevalence are dramatically reduced. If the two are the same feature, such as in the case of NDIPA, this does not impact synergistic behavior in the model, but if two different hindering features occur in the same molecule, some synergy will be observed.

Because of this, care must be taken both when applying the method and interpreting the results. While the results are useful to guide and support expert judgments, they cannot replace expert knowledge. Additionally, while the method is capable of giving compound-specific predictions of potency, it necessarily uses a simplified model of potency which is not sufficient alone to provide a reliable potency assessment for individual compounds; it does however reliably create categories that can be used to suggest analogues for AI development.

An effort was made to keep assumptions about the impact of any given feature minimal; despite this, in some cases, the magnitude of a predicted effect is still dependent on our prior assumptions. A balance must be struck between broad assumptions allowing the data (with its limitations of noise and small sample sizes) to guide predictions, versus an assessment of “what is reasonable”. The results presented here have tended toward setting broad priors, letting the data guide the predicted feature effects. Although this is a subjective judgment, the fact that the majority of predictions remain unchanged over a wide range of prior estimates, and the good agreement with blinded predictions, gives some confidence that this choice is not biasing the results.

### Prediction of Potency Categories

The use of the Bayesian model allows the probabilistic interpretation of structural features for nitrosamines and leads to the observation that, contrary to widely held assumptions that all nitrosamines are as potent as NDEA and NDMA, the majority of nitrosamine features lead to lower potencies than these two compounds by a statistically significant factor. A further set of features then lead to lower potencies than the featureless nitrosamine by another statistically significant jump. The conclusion that must be drawn from this double jump—each equivalent to about an order of magnitude in size—is that the potency-reducing features should be taken as evidence that read-across from NDEA or NDMA is inappropriate for nitrosamines that contain these potency-reducing features (isopropyl, *tert*-butyl or aromatic groups) and lack potency-increasing features; the balance of evidence is that nitrosamines with these features or carboxylic acid groups—or those with similar pharmacokinetic properties—having negative carcinogenicity results should not be surprising. On the other hand, any nitrosamine with an ethyl or methyl group, as well as benzylic, allylic, or β-carbonyl groups, should be considered likely to be positive and potentially potent. This combination of effects stresses the importance of expert review, especially in cases where a nitrosamine contains features from both lists. These lists can be seen in Table 6 and have been visualized as the graphical abstract.

**Table 6:** Substituents with Significant Effects on Potency and/or Prevalence

potency/prevalence-reducing substituents	potency/prevalence-increasing substituents
isopropyl group	ethyl/methyl
*tert*-butyl group	benzylic
aromatic group	allylic/propargylic
carboxylic acid anywhere in molecule	β-carbonyl or similar
strong β-EWGs such as CF_3_	

It should also be stressed that, due to the importance of metabolic activation, these feature lists only apply to the dialkyl/aryl nitrosamines, and not to nitrosoureas, nitrosocarbamates, or others, and apply only in the case where there is no additional toxicophore present in the molecule.

Application of these categories to determine whether nitrosamines are of highest, medium, or lower concern (using logarithmic intervals derived from the general TTC,^62^ i.e., low potency expected to be TD_50_ > 1.5 mg/kg/day, medium in the range [0.15, 1.5], and high potency < 0.15 mg/kg/day, a category with an effective lower bound of the class-specific limit (corresponding to 0.018 mg/kg/day)) gives a decision method that, if expert review is applied to those compounds with features in both lists, is either accurate or conservative with three exceptions, discussed below. The method is simple and transparent:

- Features from both lists in Table 6: Medium potency
- Concern-increasing substituent(s) only: High potency
- Concern-reducing substituent(s) only: Low potency
- No features from Table 6: Medium potency

**Figure 7:**
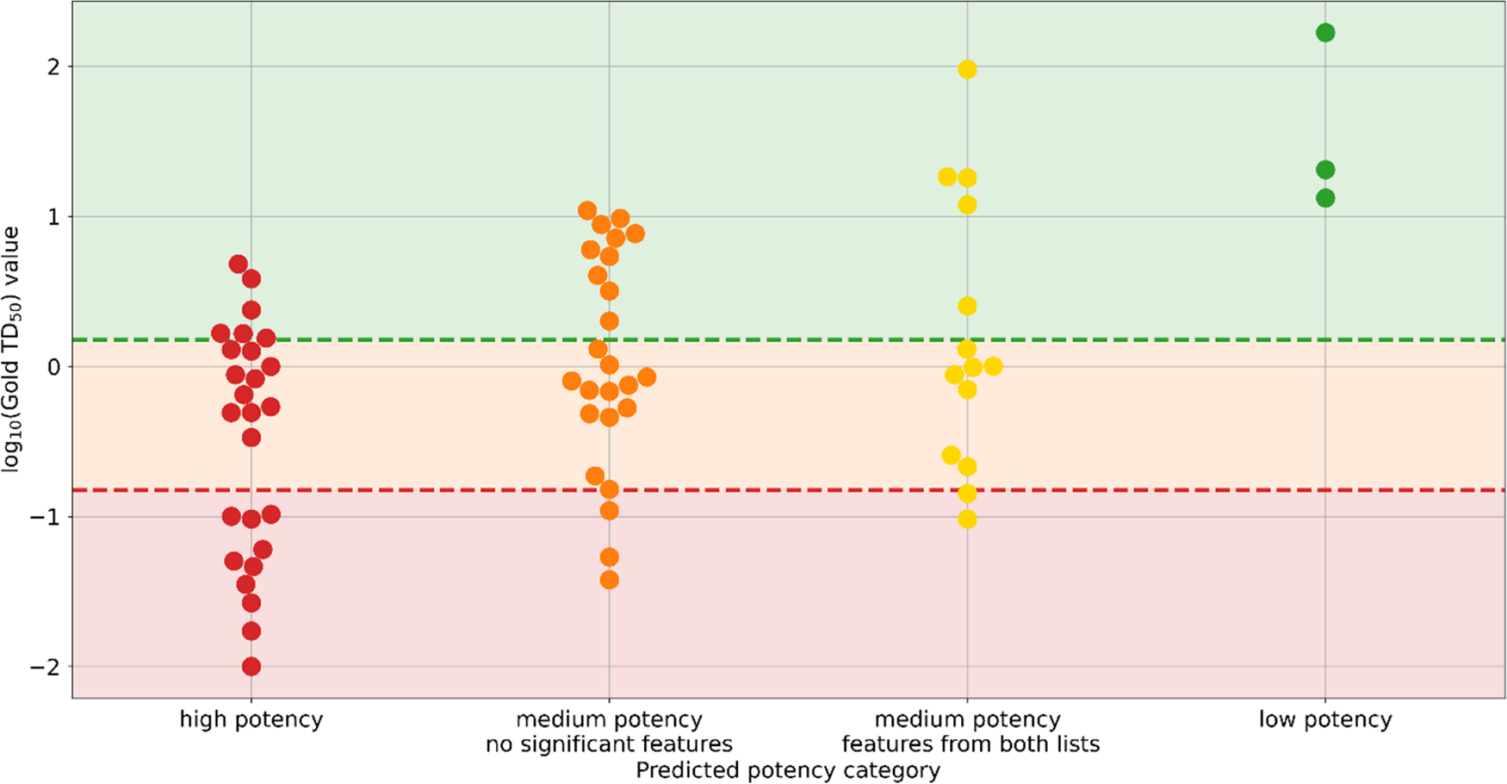
Application of the predictive model described to carcinogenic compounds with Gold TD_50_ data. Compounds have been categorized by the presence of the potency increasing and decreasing features identified using the Bayesian model given in Table 6.

The results of this method are visualized in Figure 7, and the full assignments for each compound with carcinogenicity data reported in the Lhasa carcinogenicity database are in the Supporting Information. From this, it will be seen that the majority of review-requiring compounds fit into the medium- or low-potency categories. However, since they contain features of concern such as ethyl or methyl groups, which are associated with statistically significant increases of potency and/or prevalence when considered in isolation, it was not considered appropriate to group these with compounds that contain no features of statistically significant impact, categories are kept separate.

It may be noted from the above that no provision is made for nitrosamines which are expected to be negative for carcinogenicity, via a negative Ames test or other ICH M7-compliant methodology such as the use of two contrasting QSARs. While this model is designed to be used for assigning potency for carcinogenic compounds, the features that lead to a nitrosamine being negative for carcinogenicity are implicitly captured in the use of the list of least-concerning features. Therefore, the nitrosamines that are negative in reliable carcinogenicity studies would be expected to fall into the low-potency category, the lower bound of which is the general TTC, which corresponds to a TD_50_ of 1.5 mg/kg/day, as a worst-case scenario, though for compounds not expected to be carcinogenic even this is of course exceptionally conservative.

Predictive performance statistics can also be elucidated for the model described, using Kendall’s tau coefficient.^61^ These compare favorably to the freely available carcinogenicity prediction tool Oncologic (version 9.0),^63^ which uses a comparable mechanism of reasoning between structural features, based for nitrosamines on the work of Lijinsky and co-workers.^12^ Nine of the 68 nitrosamines in the regression dataset were out of scope for Oncologic, and a further 7 were reported as containing substituents “of uncertain effect.” Since different numbers of categories are predicted (Oncologic uses six categories, ranging from low—“unlikely to be carcinogenic” to high which, being general and derived for all carcinogens, do not map clearly onto CoC- and TTC-related potency predictions of pharmaceutical relevance), direct graphical comparison is impossible; a figure comparable to Figure 7 for the Oncologic results for the 59 compounds which were in domain is in the Supporting Information. Both models correlate (at *p* < 0.05) with potency as expected, with *p*-values of 0.002 for our proposed method and 0.04 for Oncologic. As well as being more significant, our proposed classifications have a higher correlation at tau = 0.32 than those of Oncologic at tau = 0.21 suggesting our proposed method provides more informative estimates of potency. Furthermore, it is noteworthy that the model proposed is able to identify specific limits for each class (at least at the order of magnitude level) rather than an adjectival bracketing, has a broader domain of applicability—indeed near-universal, a transparent training set and methodology, and can be applied by eye by a skilled chemist.

**Figure 8:**
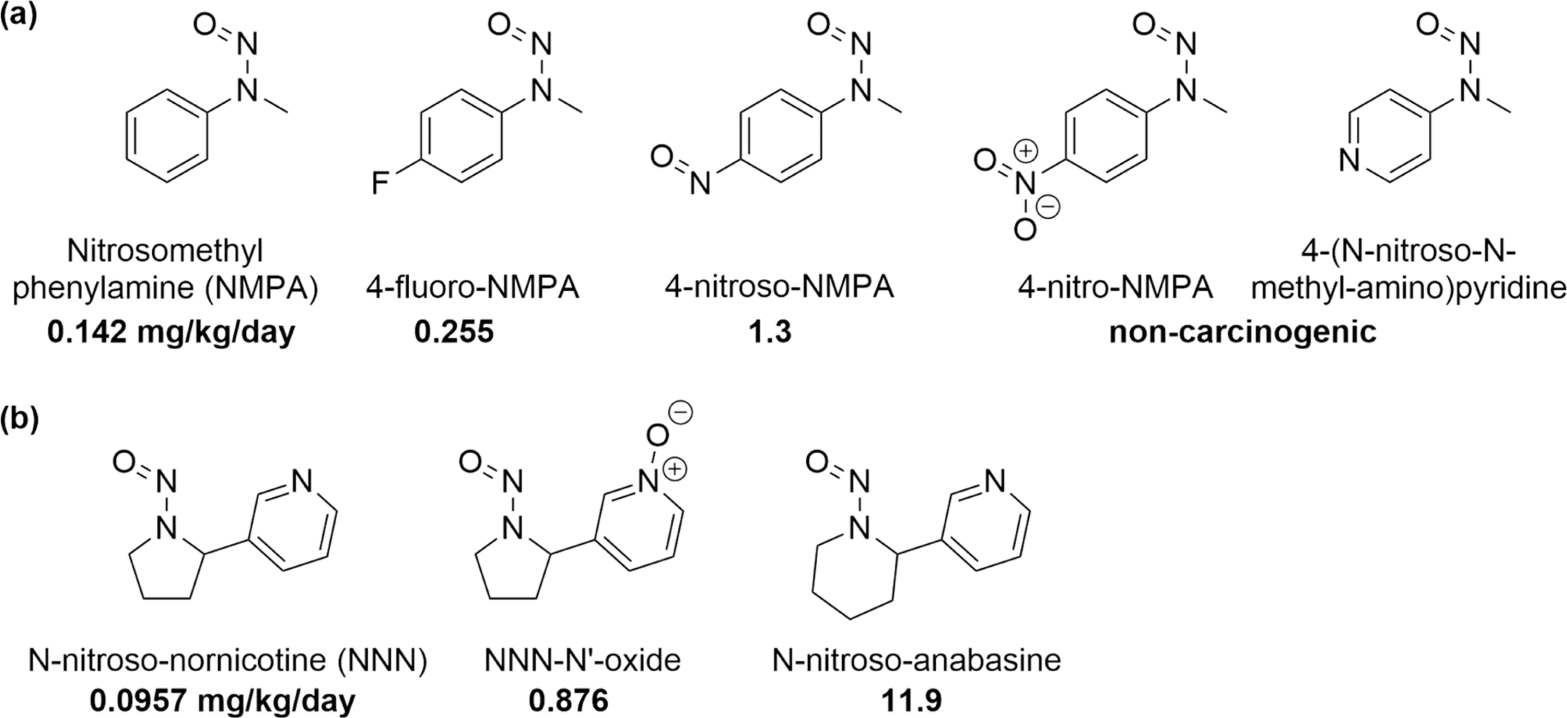
Series of compounds containing features from both lists with associated summary harmonic mean Gold TD_50_ values taken from the Lhasa carcinogenicity database.^17^ (a) Benzene and pyridine-derived *N*-nitroso-*N*-methyl aromatic amines. (b) Cyclic TSNAs.

While expert review of every prediction is important, the need for, and potential utility of, expert review for the compounds with features in both lists is stressed when the predictions for compounds with ethyl/methyl groups and aromatic groups are evaluated; this similar series of compounds has a consistent feature set but falls into all three categories, from the highly potent nitrosomethylphenylamine (NMPA) to the noncarcinogenic *N*-nitroso-*N*-methyl-4-nitroaniline.^51^ This variation would indicate a strong dependence on the electronic nature of the aromatic ring, something which is borne out looking at the full set of the *N*-nitrosomethyl-benzene and -pyridine derivatives (Figure 8a), where potency decreases as the aromatic ring becomes increasingly electron-poor—i.e., has increasingly electron-withdrawing^34^ substituents. A further category that requires expert review, and covers all three potency brackets (Figure 8b), are the TSNAs^33^ NNN, nitrosonornicotine-*N*-oxide, and nitrosoanabasine—which have aromatic substituents on the α-carbon in the nitrosamine-containing ring, which makes them both benzylic and to have isopropyl-like groups. As discussed, it has been suggested that hepatic *N*-oxidation of these is in competition with α-hydroxylation and is a detoxification route for NNN, potentially due to an increase in polarity,^59^ which would explain the decreased potency of the *N*-oxide, and that nitrosoanabasine is significantly more susceptible to this than NNN.^30,60^

Many compounds in this model are predicted conservatively, e.g., nitrosopiperazine is predicted to be of medium potency rather than low; however, due to the potential extreme carcinogenicity of some nitrosamines, a high proportion of conservative predictions was considered an acceptable outcome. It would be possible to reduce the number of conservative predictions by changing the thresholds; however, that approach has two problems: Increasing the number of compounds with underpredicted potency, which is more problematic, and overfitting to a relatively small dataset. The choice of general TTC and 10-fold lower offers a set of thresholds that are aligned with the existing regulatory environment and not fitted solely based on this dataset.

In addition to NMPA and NNN shown in Figure 8, the compounds which have no significant features yet are “unexpectedly potent”—more potent than their feature set would indicate, are shown in Table 7, as are potential reasons why the model may not predict for these compounds; all of these are compound-specific effects that may not be relevant to more complex nitrosamines. The most concerning from a potency perspective is nitrosoheptamethyleneimine; it is much more potent than nitrosohexamethyleneimine (correctly predicted as of medium potency) and may indicate potential concern for larger rings that is not able to be revealed in the available data. The feature “In a ring of size 8 or larger” is clearly not significant in its effect on potency (Figure 3), but investigation of the data shows it to be supported only by this compound. Moderately robust carcinogenicity data (multiple doses plus control, lifetime observation, although only 20 animals/sex/group) exists for this^64^ which could be used as read-across for derivatives and potentially larger rings (rather than defaulting them to 0.018 or 0.0265 mg/kg/day depending on regulatory region). Comparably, the exceptionally weak carcinogenicity of nitrosopiperazine (Table 1) is also missed since this is also the sole supporter of the feature “N_4_-unsubstituted piperazine”. Nitrosomorpholine also has a robust study,^65^ the study-specific (as opposed to harmonic mean) TD_50_ of this is 0.127 mg/kg/day—while mis-predicted, this is close enough to the category boundary as to potentially be nonsignificant compared to the variability of biological processes. In addition, this robust study can be used for read-across, although it is worth noting that some of the metabolic transformations of nitrosomorpholine^35^ may be of less relevance to its derivatives, which are typically of lower potency—hence the mis-prediction. Finally, *N*-nitroso-2,3-hydroxypropyl-(2-hydroxypropyl)amine is a hydroxylated nitrosodipropylamine derivative with a single-dose carcinogenicity experiment in a comparative study. It is clearly more potent than the other derivatives in the study,^66^ but the reasons for that potency difference are unclear,^66^ especially in light of observed low potency of the close analogue *N*-nitroso-2,3-hydroxypropyl-(2-hydroxyethyl)amine, which differs only in having a 2-hydroxyethyl group rather than 2-hydroxypropyl.

**Table 7:**
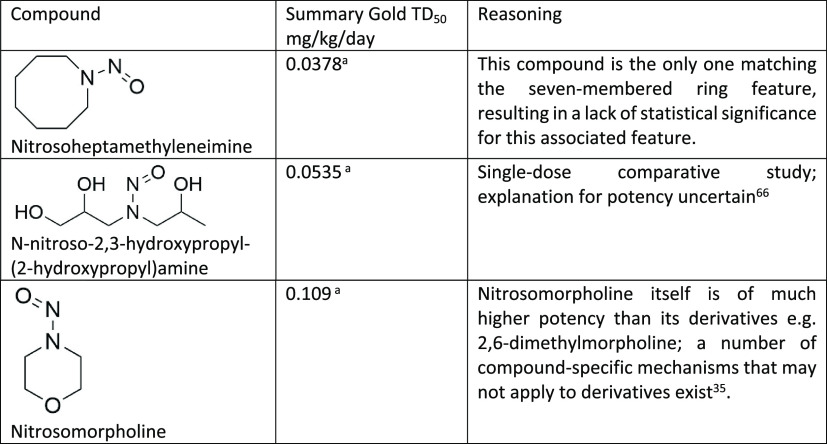
Compounds with Potency Underpredicted by the Model Described

a All three compounds were predicted to be of medium potency but were in fact high potency.

## Conclusions

A method has been developed to determine independently which structural features, from a dense overlapping set, affect the carcinogenic potency of nitrosamines. This allows the attribution of statistically significant changes in potency to certain structural features (Table 6), in close accordance with expert analysis. The predictions from this model are in some cases different from those of other (Q)SAR models that do not account for the dependence between features, such as the naïve model initially described; however, the small size of the dataset means that those models may be affected by selection bias as in the case described of the nitrosopiperidines used to study steric hindrance. The use of this method allows for analysis of feature impact without being distracted by this selection bias.

This novel synthesis of expert understanding and statistical rigor can be used to develop methods for the assessment of nitrosamine potency that, while still requiring expert review, can be used to determine recommended potency brackets for those nitrosamines that are categorized as Class 2 or 3 mutagenic impurities under ICH M7 but do not have close analogues with carcinogenicity data which can be used to set limits via read-across. The method is limited by the availability of data, as shown in the cases of nitrosoheptamethyleneimine and nitrosopiperazine, but where a compound falls into well-populated structural features shows excellent predictivity.

This method could potentially also be extrapolated to other reactive toxicophores—e.g., the genotoxicity of aromatic amines—with the proviso that the feature set must be chosen by an expert in SAR to cover the full diversity of chemical space that may surround the toxicophore with features that are (as far as possible) independent of each other.

## Supporting Information Available

Dataset with most potent summary rodent TD_50_ from the LCDB and overall carcinogenicity call (XLSX)
Full versions of Tables 3 and 4; analysis of Bayesian prior sensitivity and model cross-validation; comparable results to Figures 1 and Figures 4–6 for the full NOC dataset; and the underlying data of the analysis presented in Figure 7; comparable figure to Figure 7 for OncoLogic (PDF)

## Abbreviations

AI: acceptable intake
API: active pharmaceutical ingredient
CPDB: Carcinogenicity Potency Database
DP: drug product
EMA: European Medicines Agency
EWG: electron-withdrawing group
FDA: [U.S.] Food and Drug Administration
LCDB: Lhasa Carcinogenicity Database
NDEA: nitrosodiethylamine
NDIPA: nitrosodiisopropylamine
NDMA: nitrosodimethylamine
NDPhA: nitrosodiphenylamine
NMBA: nitrosomethylbutanoic acid
NMPA: nitrosomethylphenylamine
NMEA: nitrosomethylethylamine
NNN: nitrosonornicotine
NOC: *N*-nitroso compound
SAR: structure–activity relationship
SMARTS: SMILES Arbitrary Target Specification
SMILES: Simplified Molecular-Input Line-Entry System
TD_50_: the dose which induces tumors above control in 50% of dosed animals
TSNA: tobacco-specific nitrosamine
