# Supplementary material for: What Makes a Potent Nitrosamine? Statistical Validation of Expert-Derived Structure–Activity Relationships
Source: Chem Res Toxicol. 2022 Oct 27;35(11):1997–2013. doi: 10.1021/acs.chemrestox.2c00199 (PMC9682520; doi:10.1021/acs.chemrestox.2c00199)
Supplement: Supplementary file 2 — tx2c00199_si_002.pdf [file tx2c00199_si_002.pdf]

Supplementary information for

What makes a potent nitrosamine? Statistical validation of expert-derived structure activity relationships.

Robert Thomas[†]; Rachael E. Tennant[†]; Antonio Anax F. Oliveira[†]; David J. Ponting *[,†]

* corresponding author: david.ponting@lhasalimited.org

[†] Lhasa Limited, Granary Wharf House, 2 Canal Wharf, Leeds, LS11 5PS

Table of Contents

Naïve feature identification S2
- Regression (potency) significance S2
- Classification (prevalence) significance S3

Prior sensitivity S4

N-nitroso compounds (NOC) dataset S8
- Naïve Feature Significance S8
- Feature dependence S12
- Feature importance S13
- Comparison with expert predictions S15

Use as prediction model S16
- Comparison with Oncologic S19

Naïve feature identification

Tables 3 and 4 in the main manuscript show the most significant features in the nitrosamine dataset using a t-test to test for a difference in mean potency where TD50s are available and Fisher exact test to identify differences in overall carcinogenic call. The full results are given below.

Regression (potency) significance

Difference between compounds with and without each feature using a 2 sample t-test – assessed without the predictive model described. 17 features had sufficient data to run the test giving a Bonferroni corrected p-value threshold of 0.0029.

Feature	Support	Direction	p-value	Significant after Bonferroni correction
Et/Me only	3	More potent	0.012498	No
Has isopropyl	6	Less potent	0.018977	No
Piperidine	4	Less potent	0.022392	No
Has weak b-EWG	5	More potent	0.053022	No
Has a-CH2	2	More potent	0.098168	No
Ring size 6	12	Less potent	0.145844	No
Acyclic	21	More potent	0.195217	No
One C aromatic	9	Less potent	0.199665	No
Has Et/Me	28	More potent	0.206427	No
Allylic/Propargylic	6	More potent	0.454831	No
Morpholine	2	More potent	0.671025	No
N4-substituted piperazine	2	Less potent	0.811799	No
Heterosubstituted a-carbon	2	More potent	0.821304	No
Ring size 8+	2	More potent	0.839442	No
Benzylic	3	Less potent	0.895993	No
Ring size 5	5	More potent	0.947236	No
Pyrrolidine	4	More potent	0.95601	No
Both C aromatic	1	Less potent	-	No

Has strong b-EWG	1	Less potent	-	No
Carboxylic acid anywhere	1	Less potent	-	No
Ring size 4	1	Less potent	-	No
Ring size 7	1	More potent	-	No
N4-unsubstituted piperazine	1	Less potent	-	No

Table S1: P-values for all features in the potency dataset. This table expands on table 2 in the manuscript.

Classification (prevalence) significance

Difference between compounds with and without each feature using Fishers exact test – assessed without the model described. 25 features had sufficient data to run the test giving a Bonferroni corrected p-value threshold of 0.0020.

Feature	Support	Direction	p-value	Significant after Bonferroni correction
Carboxylic acid anywhere	13	Less potent	0.000609	Yes
Has tert-butyl	4	Less potent	0.003573	No
Has isopropyl	24	Less potent	0.019785	No
Has Et/Me	50	More potent	0.032018	No
Has a-CH2	7	More potent	0.067631	No
Has strong b-EWG	3	Less potent	0.156232	No
Pyrrolidine	12	Less potent	0.179293	No
Morpholine	4	Less potent	0.263005	No
Ring size 5	14	Less potent	0.344638	No
Ring size 6	41	Less potent	0.534176	No
N4-substituted piperazine	4	More potent	0.572908	No
Ring size 8+	3	More potent	0.572908	No
Et/Me only	3	More potent	0.572908	No
Acyclic	60	More potent	0.574823	No
Has weak b-EWG	20	Less potent	0.589065	No

Benzylic	9	Less potent	0.692753	No
Allylic/Propargylic	10	Less potent	0.713203	No
One C aromatic	14	Less potent	0.752186	No
Both C aromatic	1	More potent	1	No
Ring size 4	1	More potent	1	No
Heterosubstituted a-carbon	4	More potent	1	No
Ring size 7	1	More potent	1	No
Vinyl/Alkynyl	1	More potent	1	No
Piperidine	24	More potent	1	No
N4-unsubstituted piperazine	2	More potent	1	No

Table S2: P-values for all features in the carcinogenicity dataset. This table expands on table 3 in the manuscript.

Prior sensitivity

Being a Bayesian model the method presented in the main text is reliant on prior estimates of the effect of a given feature.

When predicting the effects of potency, the confidence of a feature having an effect on the potency is consistent for all features across a wide range of priors. Similarly, the magnitude of most features is consistent across a wide range with some exceptions. For ring features *piperazine* (both N4 substituted and unsubstituted) appears dependent on the prior. Both these features have very little compound data available, with 2 examples of a substituted piperazine and only a single example of an unsubstituted. For substitution features *both C-aromatic* and *isopropyl* features are consistently predicted to reduce potency but the magnitude of the effect is dependent on the prior, likewise the *Benzylic* feature is consistently found to increase potency, but the magnitude varies with the prior (see figure S1). As with the ring features each of these has little supporting evidence forcing the model to rely more on the prior than with other features. However it should be noted that the values shown in figure S1 represent maximum likelihood estimates only and the estimates across the full range of priors tested remain within the plausible regions estimated by the model.

The classification model is substantially more sensitive to the choice of prior with most of the ring features being highly dependent on the prior (see figure S2), although like the regression model the confidence in an effect is relatively stable compared to the magnitude. In this case we have erred towards a more conservative prior which attributes minimal changes to the features. The relatively high reliance is likely due to the lack of information contained within a positive/negative call. A TD50 gives information not only on whether a compound is more potent than another but also how much so. The regression model is able to use this information when constructing estimates of the feature effect size. In contrast a positive call does not give any information on how much more likely that call was than the base rate making estimations of effect size more difficult.

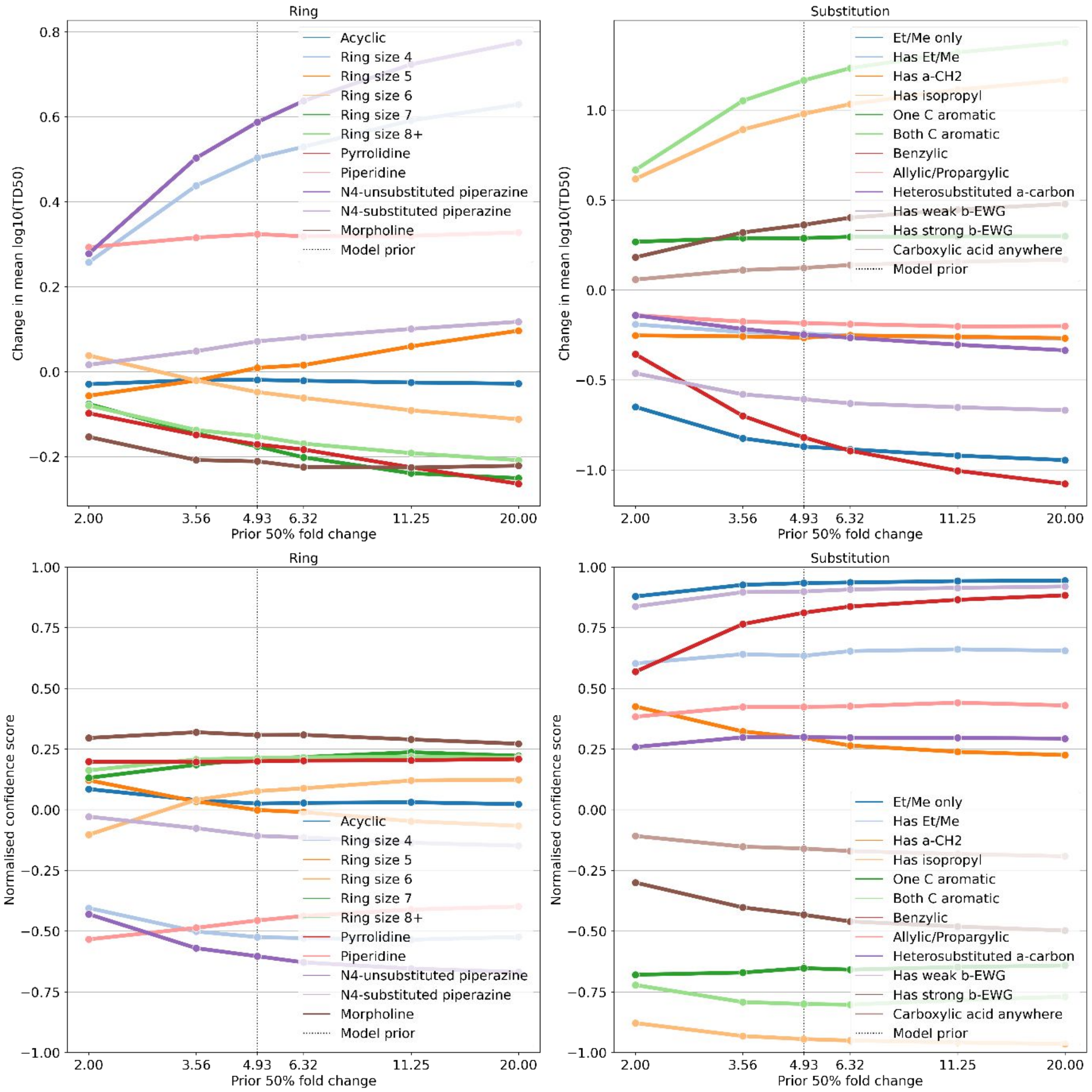

Figure S1: Maximum likelihood estimates of feature effect sizes and model confidence across a range of priors for the regression model.

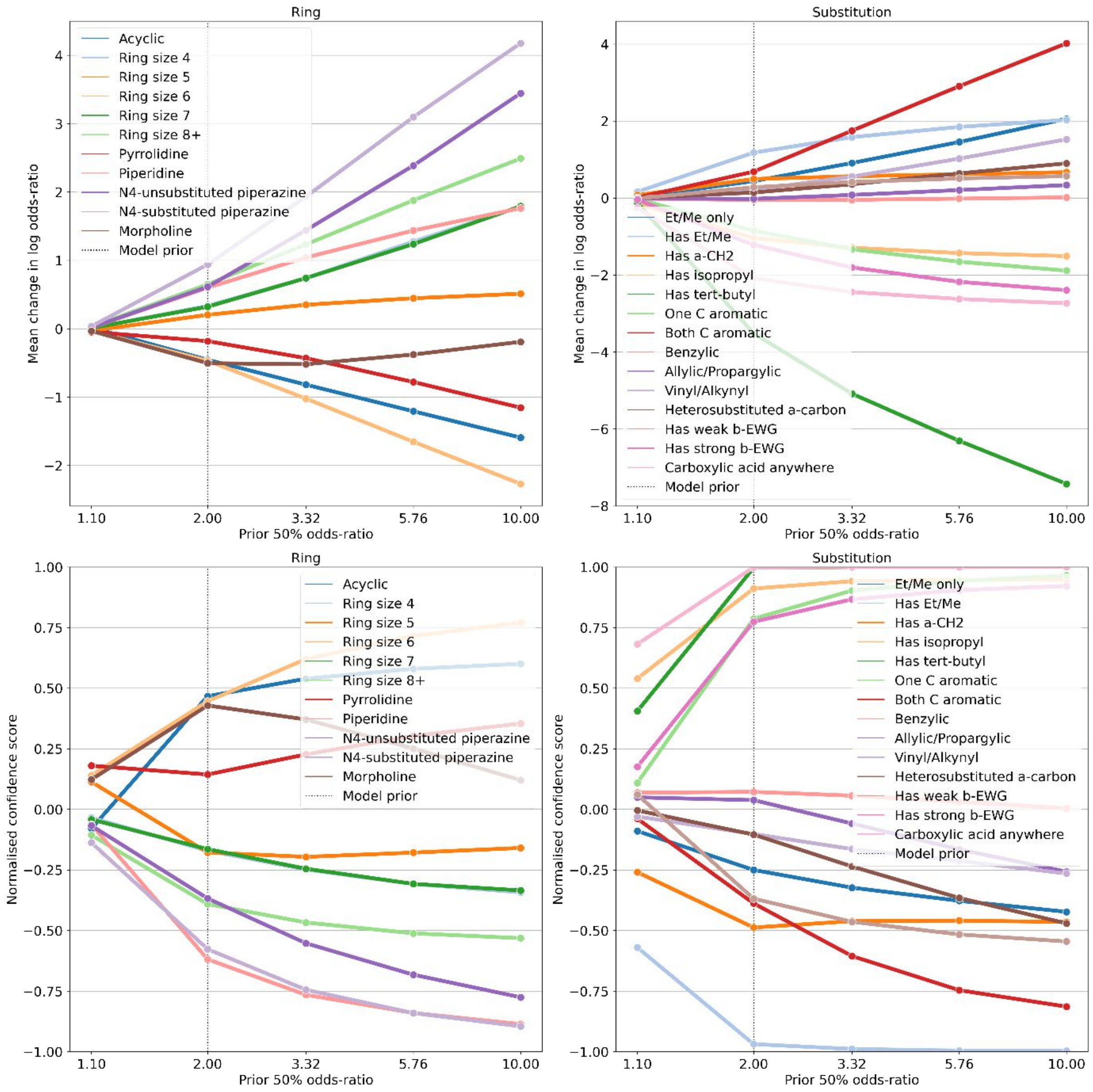

Figure S2: Maximum likelihood estimates of feature effect sizes and model confidence across a range of priors for the classification model.

Leave one out cross validation scoring[1] (LOO) was used to assess model goodness of fit across the range of priors tested. All but the most restrictive prior performed equally well for the classification task. For the regression problem tighter priors gave consistently better LOO scores, this is likely due to the fact that the TD50 distribution can be very well described by a single log-normal distribution. Tighter priors act to force the observed variation into the base distribution representing the featureless nitrosamine, as the base distribution is a very good fit this can be done without significantly decreasing the likelihood of the observations, LOO scoring applies a penalty to account for the effective extra degrees of freedom given by looser priors which then dominates the improvement in likelihood at larger *k*. At the limit of *k = 0* all variation in the TD50 would then be

[1] (Manuscript reference 26): Salvatier, J.; Wiecki, T. V.; Fonnesbeck, C. Probabilistic Programming in Python Using PyMC3. *PeerJ Comput. Sci.* **2016**, *2016* (4), e55. https://doi.org/10.7717/PEERJ-CS.55/FIG-7.

attributed to variation in the featureless nitrosamine. While this may be a mathematically valid description there are very strong reasons to expect the features of a nitrosamine to influence its potency, in this situation deciding priors based on the LOO alone is not likely to produce useful results and a prior was chosen to reflect expert assessment (*k = 1*, giving a 50% prior confidence of feature effects within a 4.93 fold change, and a 95% confidence of within 990 fold). However, as the regression model is relatively insensitive to the choice of prior other choices would give similar results.

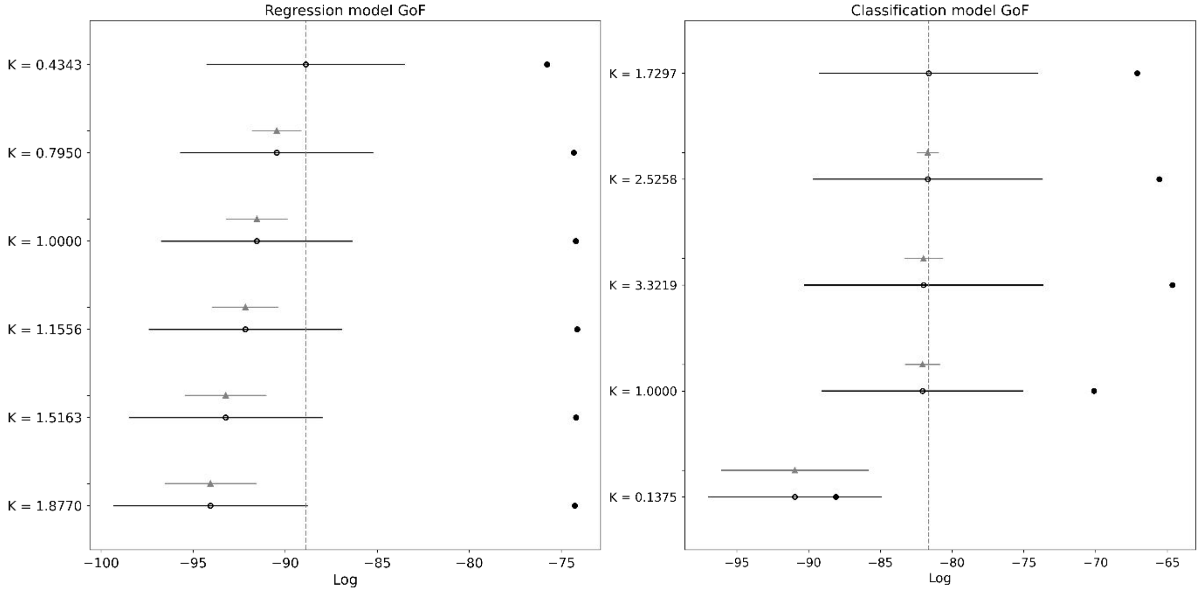

Figure S3: Goodness of fit given by model LOO scores.

N-nitroso compounds (NOC) dataset

The results presented in the main text focus specifically on dialkyl nitrosamines with other N-nitroso compounds (NOCs) being excluded from the dataset, however the same analysis has been performed on a larger set of N-nitroso compounds with the results shown here.

Naïve Feature Significance

The NOC dataset contains positive/negative calls for 231 compounds and TD50s for 112 compounds. Like the nitrosamine dataset only the *carboxylic acid* feature is significant using Fishers exact test with the Bonferroni correction, however in the regression dataset the new feature *Nitrosohydroxylamine* is significantly less potent than the dataset as a whole (see tables S3 and S4).

Feature	Support	Direction	p-value	Significant after Bonferroni correction
Nitrosohydroxylamine	3	Less potent	0.001086	Yes
Et/Me only	3	More potent	0.009155	No
Has isopropyl	6	Less potent	0.011565	No
Piperidine	4	Less potent	0.013696	No
Ring size 6	15	Less potent	0.019914	No
Has a-CH2	4	More potent	0.024476	No
Acyclic	27	More potent	0.029348	No
Has weak b-EWG	12	More potent	0.049088	No
One C aromatic	10	Less potent	0.079239	No
Has Et/Me	42	More potent	0.086031	No
Nitrosocarbamate	6	More potent	0.121814	No
Nitrosourea	29	More potent	0.239131	No
Carboxylic acid anywhere	3	Less potent	0.582578	No
Nitrosoguanidine	3	Less potent	0.605704	No
Nitramine	3	Less potent	0.605704	No
Morpholine	2	More potent	0.672708	No

Has a-Nitrile	2	Less potent	0.719298	No
Ring size 5	8	Less potent	0.761554	No
Nitrosamine	44	Less potent	0.76338	No
N4-substituted piperazine	2	Less potent	0.773471	No
Heterosubstituted a-carbon	2	More potent	0.834133	No
Ring size 8+	2	More potent	0.853637	No
Benzylic	3	Less potent	0.857273	No
Allylic/Propargylic	8	Less potent	0.924468	No
Pyrrolidine	4	More potent	0.990299	No
Both C aromatic	1	Less potent	-	No
Has strong b-EWG	1	Less potent	-	No
Ring size 4	1	Less potent	-	No
Ring size 7	1	More potent	-	No
N4-unsubstituted piperazine	1	Less potent	-	No
Nitrosoamide	1	Less potent	-	No
Nitrosated heterosubstituted amine	1	Less potent	-	No

Table S3: P-values for all features in the NOC potency dataset.

**Feature**	**Support**	**Direction**	**p-value**	**Significant after Bonferroni correction**
Carboxylic acid anywhere	15	Less potent	0.000564	Yes
Has tert-butyl	4	Less potent	0.001543	No
Nitrosourea	38	More potent	0.001588	No
Has isopropyl	24	Less potent	0.002573	No
Nitrosamine	68	Less potent	0.004132	No

Has a-CH2	12	More potent	0.018136	No
Has Et/Me	77	More potent	0.056983	No
Pyrrolidine	12	Less potent	0.071855	No
Nitrosohydroxylamine	6	Less potent	0.100465	No
Has strong b-EWG	3	Less potent	0.106085	No
Ring size 6	45	Less potent	0.147063	No
Acyclic	69	More potent	0.158796	No
Nitrosated heterosubstituted amine	4	Less potent	0.184814	No
Morpholine	4	Less potent	0.184814	No
Nitrosoamidine	1	Less potent	0.203463	No
Nitramine	9	More potent	0.210055	No
Nitrosocarbamate	8	More potent	0.364878	No
Ring size 5	18	Less potent	0.37643	No
Benzylic	9	Less potent	0.39266	No
One C aromatic	15	Less potent	0.51372	No
N4-substituted piperazine	4	More potent	0.584675	No
Piperidine	24	Less potent	0.59266	No
Nitrosoguanidine	9	More potent	0.690441	No
Allylic/Propargylic	12	Less potent	0.713358	No
Has weak b-EWG	28	Less potent	0.807381	No
Both C aromatic	1	More potent	1	No
Nitrosoamide	2	More potent	1	No
Vinyl/Alkynyl	1	More potent	1	No
Has a-Nitrile	2	More potent	1	No
N4-unsubstituted piperazine	2	More potent	1	No

Heterosubstituted a-carbon	4	Less potent	1	No
Ring size 8+	3	More potent	1	No
Ring size 7	1	More potent	1	No
Ring size 4	1	More potent	1	No
Et/Me only	3	More potent	1	No

Table S4: P-values for all features in the NOC carcinogenicity dataset.

Feature dependence

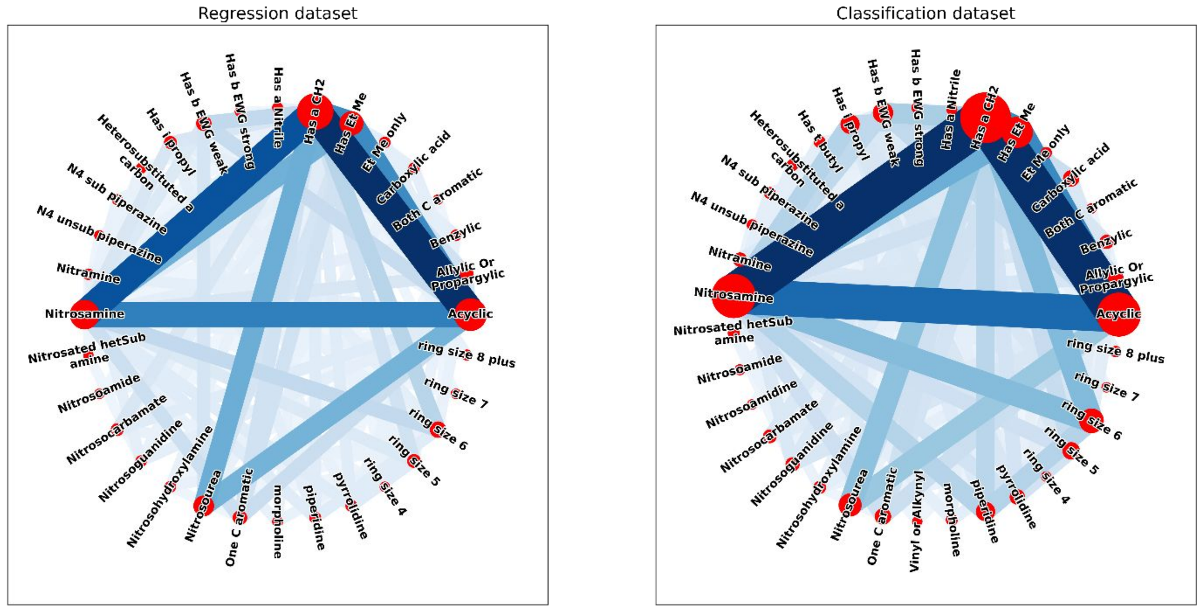

Figure S4: Overlap in compounds sharing features. This recreates figure 1 in the manuscript for the NOC dataset.

Feature importance

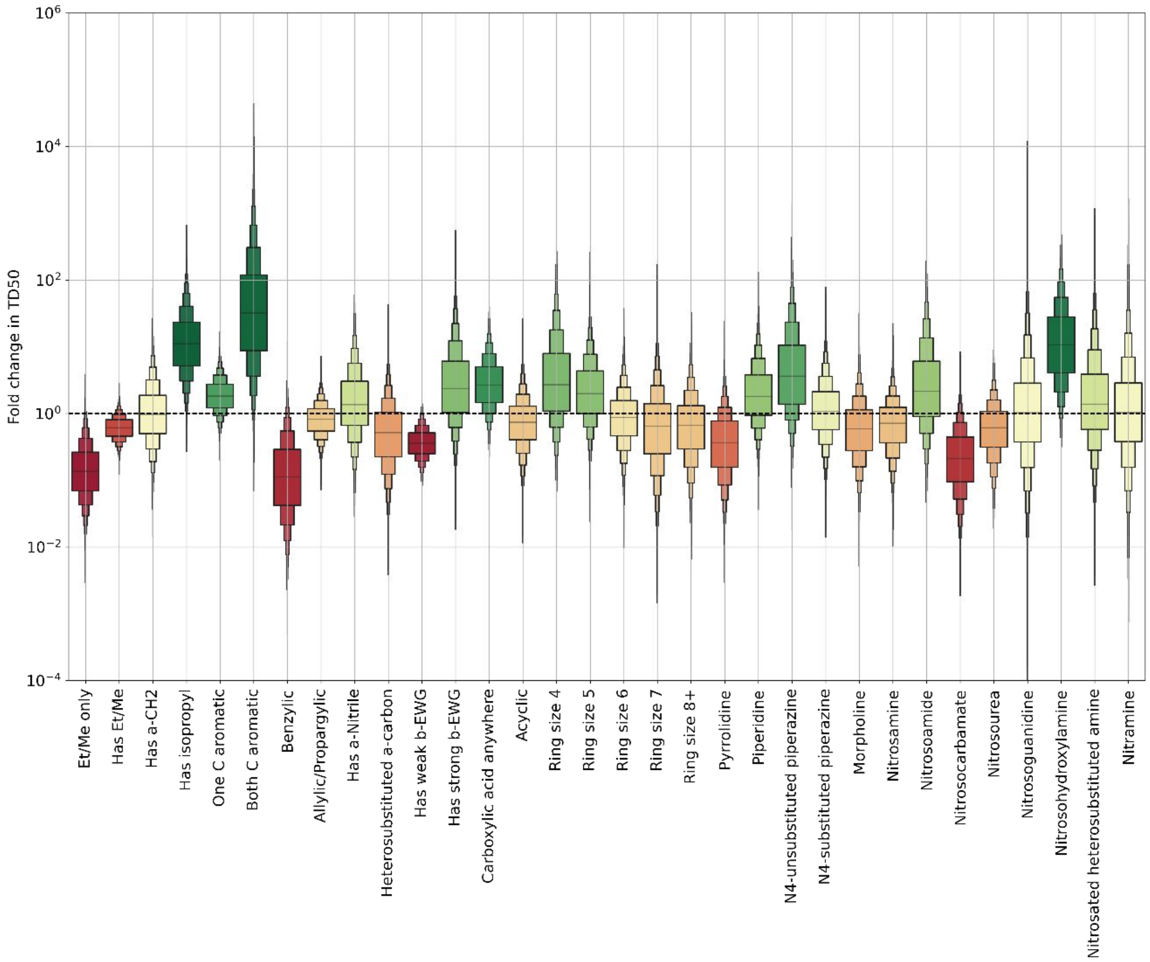

Figure S5: Predicted feature effects for the regression model based on all NOCs. This recreates figure 4 in the manuscript for the NOC dataset.

Figure S6:

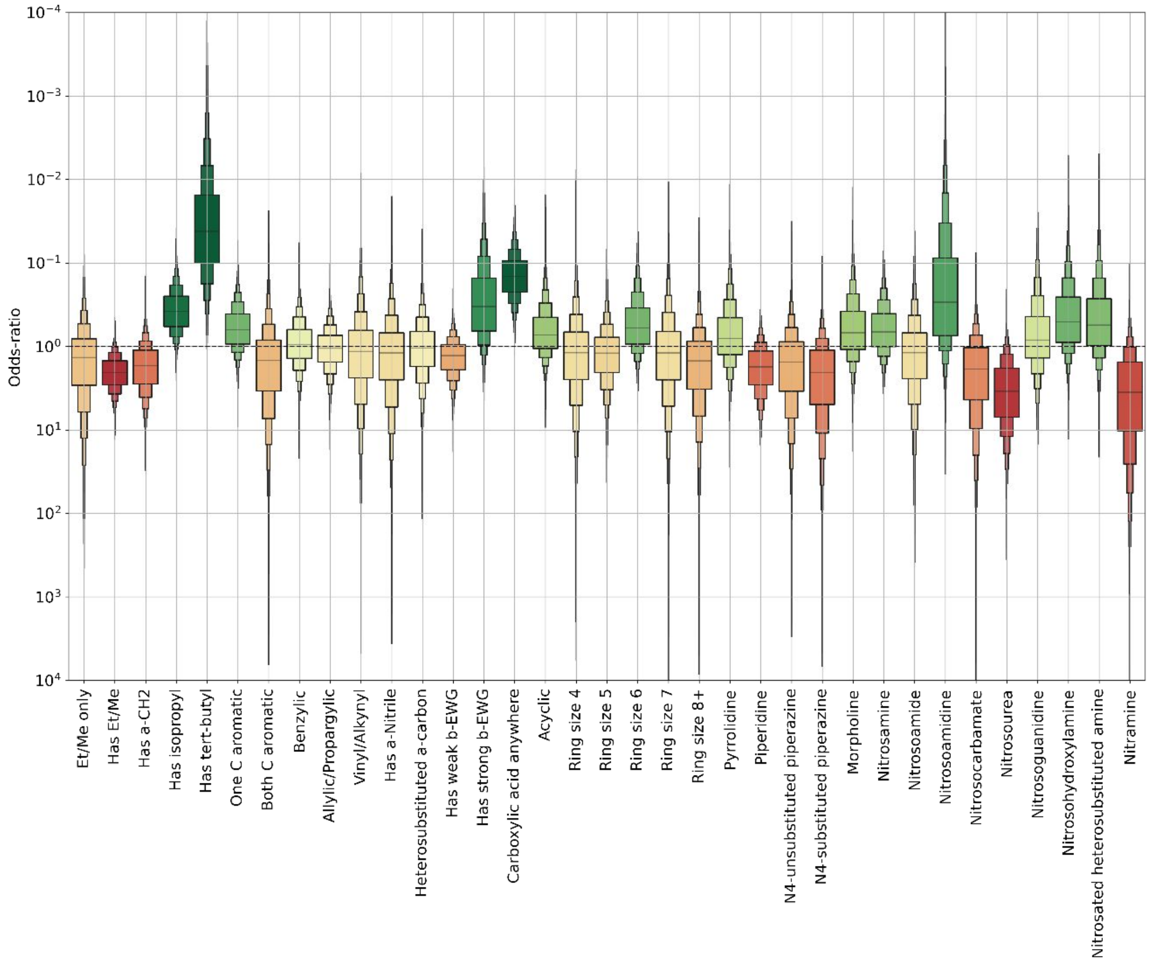

Figure S6: Predicted feature effects for the classification model based on all NOCs. This recreates figure 5 in the manuscript for the NOC dataset.

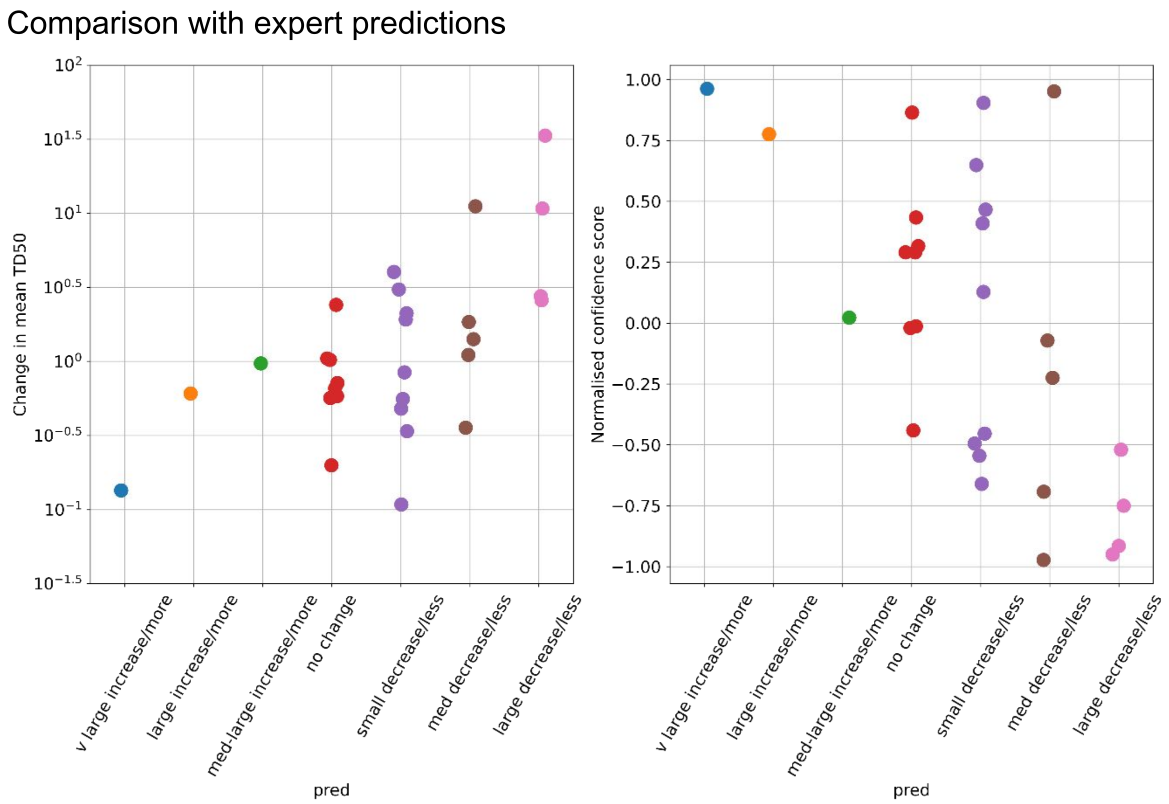

Figure S7: Comparison between expert predictions and model output for all N-nitroso compounds. Expert predictions were for nitrosamine compounds only and may not generalise to the larger chemical space, however good agreement is still found. This recreates figure 6 in the manuscript for the NOC dataset.

Use as prediction model

CAS RN®	True label	Predicted label	Concerning feature(s)	Mitigating feature(s)	TD50	Outcome
**92177-50-9**	high potency	high potency	Weak β-EWG		0.0352	**Correct**
**13256-11-6**	high potency	high potency	Has Et/Me		0.00998	**Correct**
**76014-81-8**	high potency	high potency	Has Et/Me		0.103	**Correct**
**64091-91-4**	high potency	high potency	Has Et/Me		0.0999	**Correct**
**75411-83-5**	high potency	high potency	Has Et/Me		0.0463	**Correct**
**55556-92-8**	high potency	high potency	Allylic/Propargylic		0.0601	**Correct**
**55984-51-5**	high potency	high potency	Has Et/Me, Weak β-EWG		0.0172	**Correct**
**10595-95-6**	high potency	high potency	Et/Me only, Has Et/Me		0.0503	**Correct**
**62-75-9**	high potency	high potency	Et/Me only, Has Et/Me		0.0959	**Correct**
**55-18-5**	high potency	high potency	Et/Me only, Has Et/Me		0.0265	**Correct**
**20917-49-1**	high potency	medium potency			0.0378	**Under-predicted**
**89911-79-5**	high potency	medium potency			0.0535	**Under-predicted**
**59-89-2**	high potency	medium potency			0.109	**Under-predicted**
**53759-22-1**	high potency	features from both lists	Benzylic	Has isopropyl	0.0957	**Expert review**
**614-00-6**	high potency	features from both lists	Has Et/Me	One C aromatic	0.142	**Expert review**
**86451-37-8**	medium potency	high potency	Has Et/Me		0.646	**Conservative**
**60599-38-4**	medium potency	high potency	Weak β-EWG		0.491	**Conservative**
**92177-49-6**	medium potency	high potency	Weak β-EWG		0.997	**Conservative**
**75881-22-0**	medium potency	high potency	Has Et/Me		1.26	**Conservative**
**55090-44-3**	medium potency	high potency	Has Et/Me		0.537	**Conservative**
**91308-71-3**	medium potency	high potency	Weak β-EWG, Allylic/Propargylic		0.335	**Conservative**
**91308-69-9**	medium potency	high potency	Allylic/Propargylic		0.491	**Conservative**
**88208-16-6**	medium potency	high potency	Allylic/Propargylic		0.825	**Conservative**
**26921-68-6**	medium potency	high potency	Has Et/Me		1.29	**Conservative**
**91308-70-2**	medium potency	high potency	Allylic/Propargylic		0.877	**Conservative**

**81795-07-5**	medium potency	medium potency			0.483	**Correct**
**75881-18-4**	medium potency	medium potency			0.151	**Correct**
**930-55-2**	medium potency	medium potency			0.679	**Correct**
**39884-52-1**	medium potency	medium potency			0.798	**Correct**
**932-83-2**	medium potency	medium potency			0.528	**Correct**
**53609-64-6**	medium potency	medium potency			0.846	**Correct**
**924-16-3**	medium potency	medium potency			0.691	**Correct**
**3817-11-6**	medium potency	medium potency			0.457	**Correct**
**83335-32-4**	medium potency	medium potency			0.748	**Correct**
**75896-33-2**	medium potency	medium potency			1.02	**Correct**
**621-64-7**	medium potency	medium potency			0.186	**Correct**
**100-75-4**	medium potency	medium potency			1.3	**Correct**
**61445-55-4**	medium potency	features from both lists	Has Et/Me	Carboxylic acid	0.982	**Expert review**
**145438-96-6**	medium potency	features from both lists	Has Et/Me	One C aromatic	1	**Expert review**
**16219-98-0**	medium potency	features from both lists	Has Et/Me	One C aromatic	0.214	**Expert review**
**937-25-7**	medium potency	features from both lists	Has Et/Me	One C aromatic	0.255	**Expert review**
**78246-24-9**	medium potency	features from both lists	Benzylic	Has isopropyl	0.876	**Expert review**
**16699-10-8**	medium potency	features from both lists	Has Et/Me	One C aromatic	0.699	**Expert review**
**99-80-9**	medium potency	features from both lists	Has Et/Me	One C aromatic	1.3	**Expert review**
**66398-63-8**	low potency	high potency	Has Et/Me		4.8	**Conservative**
**23834-30-2**	low potency	high potency	Has Et/Me		3.83	**Conservative**
**68107-26-6**	low potency	high potency	Has Et/Me		2.37	**Conservative**
**75881-20-8**	low potency	high potency	Has Et/Me		1.65	**Conservative**
**70415-59-7**	low potency	high potency	Has Et/Me		1.66	**Conservative**
**16338-97-9**	low potency	high potency	Allylic/Propargylic		1.54	**Conservative**

**26541-51-5**	low potency	medium potency			5.39	**Conservative**
**5632-47-3**	low potency	medium potency			8.78	**Conservative**
**15216-10-1**	low potency	medium potency			7.14	**Conservative**
**56222-35-6**	low potency	medium potency			7.65	**Conservative**
**1456-28-6**	low potency	medium potency			2	**Conservative**
**89911-78-4**	low potency	medium potency			5.98	**Conservative**
**40580-89-0**	low potency	medium potency			10.9	**Conservative**
**61034-40-0**	low potency	medium potency			9.66	**Conservative**
**13256-06-9**	low potency	medium potency			4.03	**Conservative**
**1116-54-7**	low potency	medium potency			3.17	**Conservative**
**21928-82-5**	low potency	features from both lists	Has Et/Me	One C aromatic	18.3	**Expert review**
**NoCAS-2163**	low potency	features from both lists	Has Et/Me	One C aromatic	18	**Expert review**
**82018-90-4**	low potency	features from both lists	Has Et/Me	Strong β-EWG	2.52	**Expert review**
**17608-59-2**	low potency	features from both lists	Has Et/Me	Has isopropyl	95.2	**Expert review**
**1133-64-8**	low potency	features from both lists	Benzylic	Has isopropyl	11.9	**Expert review**
**36702-44-0**	low potency	low potency		Has isopropyl	13.2	**Correct**
**14026-03-0**	low potency	low potency		Has isopropyl	20.4	**Correct**
**86-30-6**	low potency	low potency		One C aromatic, Both C aromatic	167	**Correct**

Table S5: Outcomes of using the features listed in manuscript table 8 to develop a predictive model, using the general TTC (1.5 mg/kg/day) as the threshold between medium and low potency and a threshold of 0.15 mg/kg/day as the threshold between medium and high potency.

Comparison with Oncologic

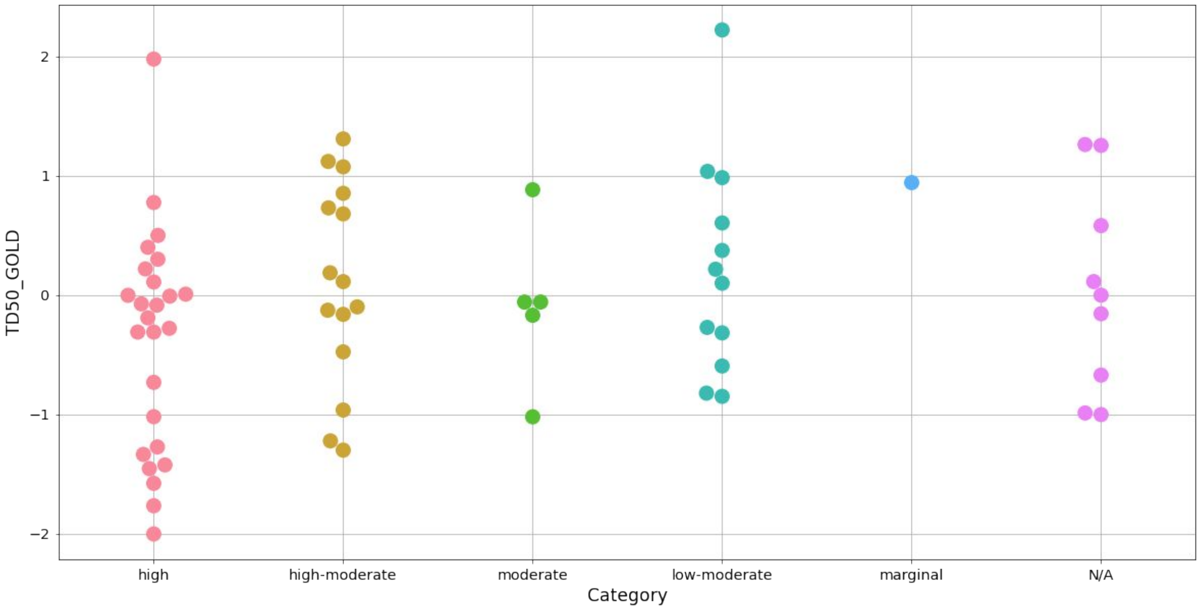

Figure S8: Predictive performance of Oncologic (U.S. EPA, version 9.0) https://www.epa.gov/tsca-screening-tools/oncologictm-expert-system-evaluate-carcinogenic-potential-chemicals for potency prediction of the nitrosamines in the dataset.
